# A Comprehensive Study of Cyanobacterial Morphological and Ecological Evolutionary Dynamics through Deep Geologic Time

**DOI:** 10.1371/journal.pone.0162539

**Published:** 2016-09-20

**Authors:** Josef C. Uyeda, Luke J. Harmon, Carrine E. Blank

**Affiliations:** 1 University of Idaho, Dept. Biological Sciences, Moscow, ID, United States of America; 2 University of Montana, Dept. Geosciences, Missoula, MT, United States of America; University of New South Wales, AUSTRALIA

## Abstract

Cyanobacteria have exerted a profound influence on the progressive oxygenation of Earth. As a complementary approach to examining the geologic record—phylogenomic and trait evolutionary analyses of extant species can lead to new insights. We constructed new phylogenomic trees and analyzed phenotypic trait data using novel phylogenetic comparative methods. We elucidated the dynamics of trait evolution in Cyanobacteria over billion-year timescales, and provide evidence that major geologic events in early Earth’s history have shaped—and been shaped by—evolution in Cyanobacteria. We identify a robust core cyanobacterial phylogeny and a smaller set of taxa that exhibit long-branch attraction artifacts. We estimated the age of nodes and reconstruct the ancestral character states of 43 phenotypic characters. We find high levels of phylogenetic signal for nearly all traits, indicating the phylogeny carries substantial predictive power. The earliest cyanobacterial lineages likely lived in freshwater habitats, had small cell diameters, were benthic or sessile, and possibly epilithic/endolithic with a sheath. We jointly analyzed a subset of 25 binary traits to determine whether rates of trait evolution have shifted over time in conjunction with major geologic events. Phylogenetic comparative analysis reveal an overriding signal of decreasing rates of trait evolution through time. Furthermore, the data suggest two major rate shifts in trait evolution associated with bursts of evolutionary innovation. The first rate shift occurs in the aftermath of the Great Oxidation Event and “Snowball Earth” glaciations and is associated with decrease in the evolutionary rates around 1.8–1.6 Ga. This rate shift seems to indicate the end of a major diversification of cyanobacterial phenotypes–particularly related to traits associated with filamentous morphology, heterocysts and motility in freshwater ecosystems. Another burst appears around the time of the Neoproterozoic Oxidation Event in the Neoproterozoic, and is associated with the acquisition of traits involved in planktonic growth in marine habitats. Our results demonstrate how uniting genomic and phenotypic datasets in extant bacterial species can shed light on billion-year old events in Earth’s history.

## Introduction

It is difficult to overstate the importance of Cyanobacteria in shaping the long-term biological and geological history of the Earth. For one, the diversification of this group has led to the oxygenation of the atmosphere [1–3]. In the modern day, cyanobacterial species comprise a large proportion of Earth’s primary productivity [4]. Unraveling the phylogenetic history of this group is critical for understanding the acquisition and timing of novel trait innovations and determining how such events may have impacted Earth history.

Some questions remain over whether meaningful evolutionary inferences can be made over deep geologic timescales, especially due to the enormous billion-year time-spans separating extant species from their common ancestors and the potential for horizontal gene transfer [5, 6]. However, it is becoming increasingly evident that there is substantial phylogenetic signal in the slowly evolving housekeeping proteins of prokaryotic cells [7–9]. Multiple studies of prokaryotic genomes show that lateral gene transfer is not as “rampant” as previously thought, particularly among the slowly evolving core housekeeping and metabolic genes [10, 11]. Indeed, measured rates of lateral gene transfer among metabolic genes show these events occur only slowly (with rates of up to 4.0 events per billion years; [12]) and that most lateral gene transfer events appear to occur between closely related taxa and among taxa that inhabit common niches [13, 14], suggesting that comparative approaches to trait evolution using phylogenetic trees might be a profitable approach in these groups.

Large amounts of sequence data are needed to construct prokaryotic relationships that are very ancient [15, 16]. As a consequence, phylogenetic reconstruction of prokaryotic evolutionary relationships often relies on the concatenation of large numbers of protein (amino acid) sequences from whole genome sequences [15, 17]. Large concatenated datasets, just like single-gene datasets, are still susceptible to phylogenetic artifacts such as lack of signal or long branch attraction [18, 19]. Indeed, many such artifacts pervade the phylogenetics literature [16], leading to erroneous inferences of early microbial trait evolution. Thus, it is imperative that phylogenetic trees of prokaryotic groups be formally tested for these artifacts in order to identify and remove them from phylogenies.

Once a robust, artifact-free backbone phylogeny is available, phylogenetic comparative methods (such as ancestral state reconstruction) can be used to study the evolution of phenotypic traits (such as metabolic, morphological, and ecological characters). These methods have been widely applied to understanding the evolutionary history of macroscopic eukaryotes, but only rarely applied to prokaryotes (although see [18–26].

Here we resolve the cyanobacterial tree using a large concatenated genomic dataset. After demonstrating statistically significant phylogenetic signal underlying most of the traits studied, we show that particular morphological and ecological traits have been gained in discrete intervals in Earth’s history, with many of those trait acquisitions occurring in freshwater environments over a prolonged stretch of geologic record. We show that the earliest lineages were likely freshwater, had cell diameters less than 2.5 *μm*, were benthic or sessile, and possibly were epilithic/endolithic with a sheath. Later acquisitions of sheathed filamentous morphologies, larger cell diameters, and nitrogen fixation are seen in freshwater environments around the end of the Paleoproterozoic and early Mesoproterozoic. Much later acquisitions of branched morphologies, followed by marine planktonic nodes in the Neoproterozoic.

**Fig 1:**
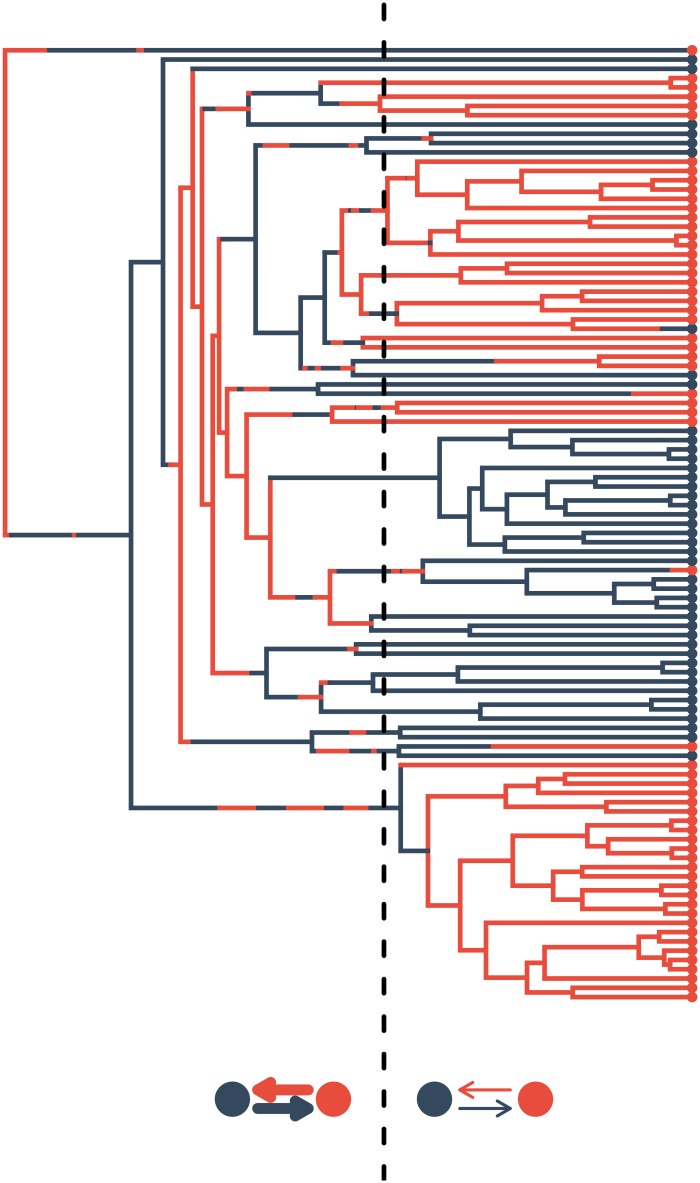
Epoch model fit to binary trait data. The epoch model describes the transition rates between traits (red and black) where a single shift at a point in time on the phylogeny results in a change in the overall transition rates. In this case, we observe higher transition rates prior to the dashed black line–followed by relative stasis.

We also looked for evidence for changing rates of trait evolution through deep time. We use “epoch models” that extend standard discrete trait models to allow for a single phylogeny-wide shift in the rate of gains and losses of binary traits (Fig 1). We find that most traits exhibit a signal of decreasing rates of gains or losses as well as relative fixation of character states (i.e. evolutionary stasis) in the wake of the Great Oxygenation Event (GOE). We also show some evidence for later rate shifts occurring near the Neoproterozoic Oxygenation Event (NOE)—suggestive of diversification of species into marine, planktonic habitats. Our approach demonstrates that important inferences regarding the dynamics of trait evolution can be derived from the billion-year old history of Cyanobacteria using phenotypic data and phylogenetic comparative methods.

## Materials and methods

**Phylogenomic dataset** Phylogenomic trees were calculated using a concatenated genomic dataset containing 137 amino acid and two ribosomal RNA sequences (large subunit and small subunit) universally found in Cyanobacteria. The protein sequences sampled a broad array of cellular functions in the core of the cell [19]. Analyses were carried out in order to reconstruct the phylogenomic tree that represents the evolutionary pattern of the core genome (in contrast to the pangenome). Genome sequences were obtained from GenBank using PBLAST (from the nr database) or tBLASTn (from the wgs database). Sixty-five new genome-sequenced taxa were added to a previously published alignment of 69 Cyanobacteria [27], for a total of 134 taxa. Ribosomal RNA sequences were manually aligned according to secondary structure in order to align homologous nucleotides [28]. Protein sequences were aligned using ClustalW and hand-edited using a text editor (TextWrangler). Character sets were defined such that only unambiguously aligned regions were used to construct the phylogenetic trees (character set included 49,749 characters, S1 File).

**Tree construction and long branch attraction identification** Phylogenetic trees were then constructed using Bayesian analyses carried out with MrBayes v. 3.1.2 and v. 3.2 [29] using the CIPRES Science Gateway (www.phylo.org) and maximum parsimony method in PAUP* v. 4.0b10 [30]. Bayesian analyses were performed using 4 chains, default priors, and two partitions. The first partition contained the amino acid sequences and the second contained the nucleic acid (rRNA) sequences. Both partitions included a gamma distribution with 3 rate categories and a GTR model for nucleic acid sequences. Analyses were run until the topology converged or the average standard deviation of split frequencies was less than 0.01.

**Fig 2:**
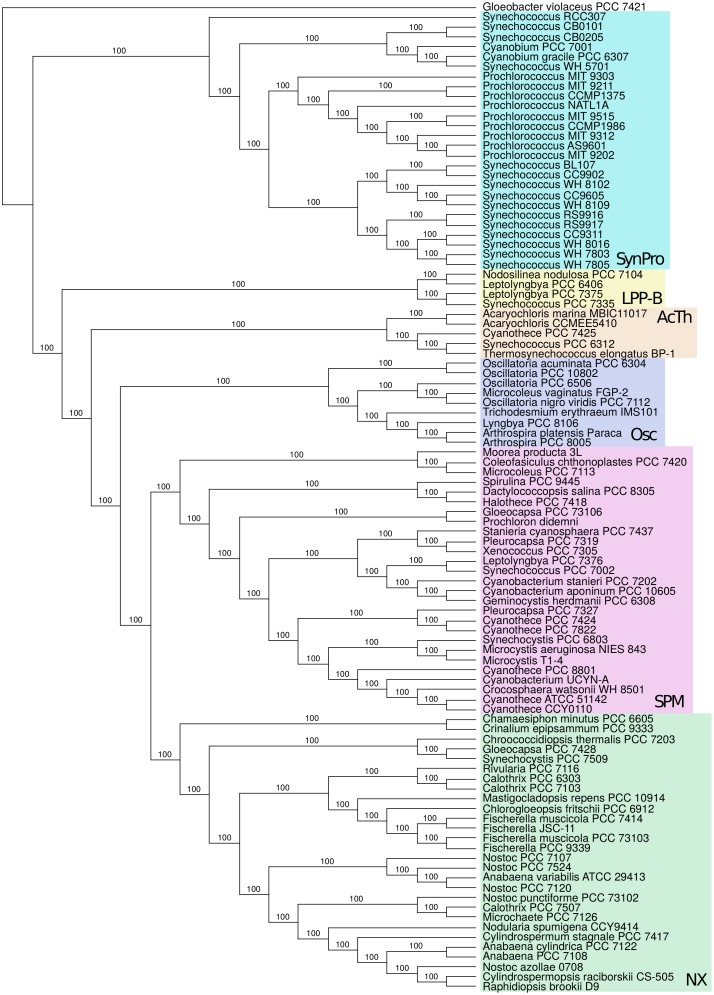
Core cyanobacterial Tree. Tree constructed using MrBayes; all branches were supported with 100% posterior probability. Clades are labeled as follows: SynPro (*Synechococcus* + *Prochlorococcus* + *Cyanobium*), LPP-B (“LPP group B”; *Leptolyngbya + Nodosilinea + Synechococcus*), AcTh (*Acaryochloris + Thermosynechococcus*), Osc (*Oscillatoriales sensu stricto*), SPM (*Synechocystis + Pleurocapsa + Microcystis*), NX (*Nostocales sensu lato* + others).

**Fig 3:**
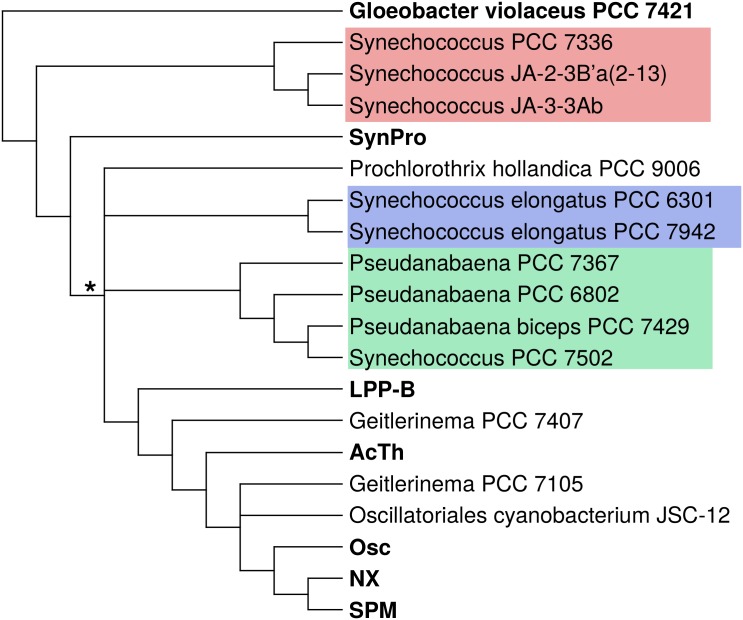
Composite Tree Showing Branching Location of Rogue Taxa. This consensus tree summarizes the results of the rogue taxa addition and subtraction experiments. Lineages in bold indicate clades found in the core tree. The * indicates the clade of taxa that contain an intron in the tRNA-Leu gene and an intein in the DnaE protein. Distinct clades of rogue taxa are indicated with colored boxes: red shows the Octopus Spring clade, blue the Synechococcus elongatus clade, and green the Pseudanabaena clade.

Taxon addition and subtraction experiments allowed the identification and avoidance of long branch attraction artifacts (LBA; [18, 19]). These analyses revealed tree construction with most cyanobacterial taxa resulted in a well-resolved core phylogenetic tree (“core taxa”, Fig 2). They also revealed that several taxa were highly susceptible to LBA artifacts (here called “rogue taxa”; Fig 3).

Because a single tree could not be resolved that contained all taxa (without inducing LBA artifacts), two sets of tree files were therefore generated. The first set of 9 tree files (each containing 100 MrBayes trees sampling the posterior probability distribution of tree topology and branch lengths) included the core taxa plus different samplings of the “rogue taxa”, and lacked any (known) branching artifacts. The second set of 3 tree files (also sampling the posterior distribution) included the core taxa plus different samplings of the “rogue taxa” and did contain known branching artifacts. These trees were analyzed to determine how trees with known LBA artifacts affected ancestral state reconstruction or inferences made using the epoch model.

**Ancestral state reconstructions** We coded a large number of binary character states from taxonomic descriptions of Cyanobacteria in the published literature, using Mesquite v. 3.01 [31]. Characters coded included thermophilic, habitat salinity (freshwater, brackish, marine, hypersaline), akinetes, heterocysts, nitrogen fixation, morphology (unicellular, filamentous/pseudofilamentous), polarity of filaments (isopolar, heteropolar), growth habit (planktonic, sessile/benthic), growth relationships (free-living, epilithic, epiphytic, epizoic, periphytic, endophytic, endolithic, presence in microbial mats), motility, motility type (gliding, rotational), hormogonia, gas vesicles, false branching, true branching, fission in multiple planes, trichome type (uniseriate, multiseriate, multitrichomous), baeocytes, extracellular sheath, and mucilage. Most characters were coded as presence/absence (0 = absence, 1 = presence), however characters with multiple character states were coded in two different ways (as single characters with multiple character states and as multiple characters with binary character states—i.e. presence/absence). Cell diameter data (minimum cell diameter, average cell diameter, and maximum cell diameter) were coded as continuous characters. Cell diameter data was obtained from taxonomic descriptions in the primary literature, from the Pasteur Culture Collection of cyanobacteria, or from Bergey’s manual [32]. When cell diameter data for the strain or species could not be found, the cell diameter for the genus was used. Average cell diameter was also coded as a binary discrete character (0 = less than 2.5 *μm*, 1 = equal to or above 2.5 *μm*). Ancestral state reconstruction (ASR) was performed using maximum parsimony (for ordered multistate traits, such as habitat salinity, because many taxa have multiple character states such as “brackish and marine”) and maximum likelihood using the Mk1 model (for binary traits) in Mesquite.

**Age constraints and age estimates of major clades** A number of age constraints were implemented to estimate the potential age of nodes in the tree. The cyanobacterial root minimum age was set at 2.32 Ga (the rise in atmospheric oxygen at 2.32 Ga according to sulfur isotopic fractionation patterns and carbonate-associated iodine; [33, 34]. The root maximum age was set as two different times: 3.8 Ga (the end of late heavy bombardment, a very conservative setting given current large uncertainties as to when Cyanobacteria originated) or 2.7 Ga (what is generally interpreted as possible localized oxidative weathering in an otherwise anoxic atmosphere; [35]). A number of fossilized traits (akinetes, large cell diameters, unbranched and branched filaments) associated with Cyanobacteria have been identified in the rock record. These were used to construct age constraints for relaxed clock analyses.

Heterocysts and akinetes—thick-walled resting stages reported in the fossil record—are differentiated cells found in the Nostocales and Stigonematales (also referred to as Stigonemataceae; [36]; Fig 2). Phylogenomic analyses show that these two orders form a monophyletic group (with Stigonematales/Stigonemataceae nested within the Nostocales), and thus they should not be considered to be two separate groups as they have in the past [37]. Howard-Azzeh et al. [37] proposed a higher clade of akinete-producing cyanobacteria within this monophyletic group, however akinetes have been reported in taxa outside of their identified clade in *Chlorogloeopsis* and *Fischerella* [38, 39] and therefore likely arose earlier in the group. ASR predicts that akinetes arose independently twice in the Nostocales-Stigonemataceace (which will be referred to here as Nostocales *sensu lato*). However, it is also possible that this is a complex trait that arose once in the ancestor to this group, and that there have been multiple losses of this trait. Ultrastructural analyses of *Anabaena cylindrica* and *Fischerella muscicola* shows similar structures in common (membrane stacks, glycogen and cyanophycin granules, and a new outer envelope/cell wall laid down outside the vegetative cell; [40]). Given these similarity of features, is may be more likely that there was a single origin of akinete structures.

In the fossil record, purported akinetes are identified as modified structures in filamentous microfossils that generally have an elongated shape and larger cell diameter than vegetative cells (as Archaeoellipsoides; [41]). Such akinete-like structures are dominant in Mesoproterozoic Kotuikan assemblages, 1.65–1.20 Ga [42]. Thus, 1.65 Ga was used as a minimum age for the akinete-bearing Nostocales. It should be noted, however, that Butterfield [43] questions that Archaeoellipsoides correspond to akinetes, and therefore some analyses did not invoke this age constraint. The ancestor to the Nostocales clade is also inferred to have had a heterocyst (S7 Fig). Although it is not known from the geologic record how old heterocysts are, these structures have been proposed to have appeared as an adaptation for fixing nitrogen in an oxygen-rich atmosphere [44]. Thus, 2.32 Ga (this rise in free atmospheric oxygen) was used as the maximum age for the Nostocales.

In addition to akinetes, cell diameter has long been used as a criterion to distinguish potential Cyanobacteria in the fossil record. Indeed, large cell diameters (above 2.5 *μm*) are considered to be characteristic of the Cyanobacteria [45]. More recently it has been shown that large cell diameters are not the ancestral character state in the Cyanobacteria [19]. Cell diameters between 2.0 and 6.5 *μm* first appeared in the fossil record at 2.0 Ga [46]. The oldest, most inclusive clade with an an inferred average cell diameter >2.5 *μm* (using ASR, see below) was constrained to have a minimum age of 2.0 Ga and a maximum age of 2.45 Ga (a time before which there is a paucity of large cell diameters).

Finally, morphologically simple (unbranched, isopolar) trichomes and sheaths are also distinct cyanobacterial morphologies noted in the fossil record. Such morphologies appear and become abundant in the fossil record starting with the Gunflint-type and Belcher-type microbiotas (1.9 Ga; [42]). The oldest, most inclusive clade with an an ancestral node inferred to be filamentous with a sheath (using ancestral state reconstruction, see below) were constrained to have a minimum age of 1.9 Ma and a maximum age of 2.45 Ga (due to a lack of sheathed filaments in older assemblages). Note that for all age constraints, only a single node was constrained, and we did not constrain younger clades that originated these traits.

After setting minimum and maximum age constraints, trees (with branch lengths calculated by MrBayes) in the 9 tree files were converted to time trees (with branch lengths scaled to time) using Penalized Likelihood in r8s version 1.7 [47, 48]. For each tree, the smoothing parameter was optimized using cross-validation and a logarithmic penalty function implemented. Trees where the branch lengths had been converted to time were then re-imported into Mesquite to be used for a second round of ancestral state reconstructions. The resulting figures combined ASR with relaxed molecular clock analysis, facilitating visualization of when in geologic time nodes with particular traits likely appeared.

**Box 1. Description of traits examined in this study**

**Akinetes.** Thick-walled specialized cells in a filament that function in dormancy and resilience.

**Baeocytes.** Small daughter cells resulting from repeated rounds of fission that may be enclosed by a sheath.

**Epi/Endolithic.** Either living on or inside rocky substrates.

**Epiphytic.** Growing on plants.

**Extracellular sheath.** Part of the cell envelope, located outside the cell wall and has a protectivei function. Often pigmented, it has a microfibrillar structure and is composed of polysaccharides/polypeptides.

**False branching.** Branching of the filament that occurs when trichomes break or fragment within a filament and break out at the location of a necridic cell or a heterocyte.

**Filament.** Comprised of a trichome and a sheath.

**Filamentous morphology.** A multi-cellular morphology where cells form part of a trichome.

**Fission in multiple planes.** Cell division in two or three perpendicular planes.

**Freeliving.** Planktonic, living unattached to any substrate.

**Freshwater habitat.** Existing in low salinity (less than 0.5% salts), terrestrial habitats.

**Gas vesicles.** Or gas vacuoles, are intracellular gas-filled proteinaceous chambers used to regulate buoyancy in the water column.

**Gliding motility.** The ability to move over a solid surface.

**Growth habit.** The physical relationship of the organism with environmental materials, such as being benthic, sessile, attached to a substrate, or planktonic.

**Heterocysts.** Thick-walled, specialized cells specialized for carrying out aerobic nitrogen fixation.

**Heteropolar filaments.** Filamentous morphology, where both apical ends of the filament have a different size or morphology.

**Hormogonia.** Short chains of motile cells, motile by means of gliding motility or gas vacuoles, which break off a larger filament and serve a role in migration or dispersal.

**Isopolar filaments.** Filamentous morphology, where both apical ends of the filament have the same size and morphology.

**Marine planktonic.** Free-living, planktonic species in marine (salinity between 3–5%) environments.

**Microbial mats.** Thick, laminated microbial structures, where different layers contain microbial taxa specialized in biogeochemical niches, which often contain Cyanobacteria in the upper-most layers that are exposed to light.

**Motility.** Movement across a surface or through a liquid medium. In the Cyanobacteria, generally comprises gliding motility, although novel forms of motility have been described in several species.

**Mucilage.** Part of the envelope outside the cell wall, generally comprised of exopolysaccharides and lacking a defined structure.

**Multiseriate trichomes.** Trichomes that form when cell division occurs both at right angles to the length of the trichome and parallel to the plane of the trichome—resulting in trichomes that have a width of more than one cell.

**Nitrogen fixation.** The ability to fix atmospheric nitrogen into ammonium.

**Periphytic.** The growth habit of living on submerged plants, animals, algae, sediment, or rock.

**Thermophilic.** Organisms that have an optimum growth temperature above 45°.

**Trichome.** A chain of cells, formed by binary fission in a single plane, where cells remain attached to one another after cell division, may or may not have a sheath.

**True branching.** Branching of the filament that occurs when cell divisions mainly occur perpendicular or obliquely to the main axis of the trichome. Branches remain physically attached to the main trichome.

**Uniseriate trichomes.** Trichomes that form when cell division occurs at right angles to the trichome, resulting in a trichome having a width of one cell.

**Unicellular.** A morphology where cells detach from one another after cell division.

**Phylogenetic signal in traits** Using the time trees, the presence of significant phylogenetic signal in coded morphological traits was determined with Pagel’s *λ* [49] using Arbor [50]. To do this, we compared the likelihood of an Mk2 model with *λ* fixed at 0 (that is, a no phylogenetic signal model) to an Mk2 model with *λ* estimated from the data using a likelihood ratio test.

**Discrete trait epoch model** Models of character evolution in binary traits allow for estimation of the rate of evolutionary transitions (gains and losses) between character states across the phylogeny (Mk models, [51–53]). These models are the basis for inference of ancestral states and closely related to models of sequence of evolution used to estimate phylogenies. We tested whether particular times in the earth’s history are associated with elevated or decreased transition rates across traits, perhaps in association with adaptive trait diversification events and/or major geologic events. To test this hypothesis, we used models that build upon the standard Mk models typically used to model discrete traits, but allows the rate of gains and losses to change at a discrete time point along the phylogeny. We thus divide the phylogeny into two “epochs” in which independent models of trait evolution are fit to each epoch [54–56]. Thus, the model has 3 additional parameters, the location of the time shift, and the change in the rate of both gains and losses after the time shift. We parameterized the model so that after the shift each rate was modified by a rate multiplier, *r*_01_ and *r*_10_, which correspond to the relative increase or decrease of the rate of gains of trait 1 and trait 0 after the time shift, respectively. We fit epoch models to each trait independently using the R package diversitree [56].

In addition to fitting each trait independently, we sought to determine whether we could detect correlated changes in the rate of character evolution across traits in the cyanobacterial phylogeny. To determine whether there was significant support for an overall shift in the model across traits, we fit a joint model in which parameters for 25 traits (a representative sample of the traits coded in this study) were estimated simultaneously with a single single shift location and rate multiplier across all traits (but allowed independent transition rate parameters for each trait). We compared this model fit to a model fit without a time-shift. This allowed us to evaluate the support for an overall shift without introducing an unreasonable number of extra parameters. We also estimated the marginal ancestral states at each node using the best-fitting epoch model for each trait.

Some traits may exhibit different patterns of evolution, so fitting a single joint model can conceal or average out these disparate patterns. Therefore, we employed an alternative strategy to look for more fine-scale patterns in the timing of shifts across traits. For each trait, we fit an epoch model for a sequence of 38 rate-shift locations at regular intervals along the height of the phylogeny. We then took the difference between the likelihood value of the best-fitting model for each shift location and the no-shift model, generating a likelihood profile across different shift locations. We then took the cumulative sum of each likelihood profile over all of the traits examined. The cumulative likelihood profile can then be examined for peaks which indicate support for a shift across traits, while simultaneously allowing us to look for concordant patterns of divergence across traits.

**Simulation study of character evolution along the cyanobacterial phylogeny** We considered the possibility that the structure of the phylogeny itself, combined with errors in either or topology or branch length estimation, could result in artifactual evidence of shifts concentrated at certain time points. To test this possibility, we designed simulations to evaluate the degree of support for the epoch-model under the null model of constant rate evolution.

We evaluated the degree to which topological error could affect our results. A random tree was drawn from the set of 9 tree files associated with different taxa sets, including those with long-branch attraction artifacts. Each tree file included 100 trees from posterior distribution, which were pooled for a total of 900 trees that could potentially be sampled by our simulations. We fit a constant-rate Mk model for each trait and phylogeny in the dataset to sample the range of realistic parameter values consistent with our dataset. For each simulation, we first randomly selected among this set of model fits to obtain the true parameters for our simulating model. We then randomly selected another tree from the posterior distribution, simulated a constant-rate Mk model on that tree, and subsequently re-estimated parameters under the epoch model using a different tree drawn from the posterior distribution. Since we drew model parameters from the set of estimated parameters for the actual empirical dataset, we were able to evaluate how branch length and topological uncertainty in the posterior distribution could influence the artifactual appearance of shifts under an epoch-model. For example, trees drawn from the same tree file generally displayed much less branch length and topological variation compared to trees drawn from different tree files and taxa sets. This allowed us to evaluate the degree to which support for the epoch model varies with model parameters, topological and branch length errors (see S1 Methods). We conducted 1000 simulations and pairings of randomly selected source tree, simulation parameters, and estimating trees.

## Results

**Core cyanobacterial tree.** Taxon addition and subtraction experiments were performed in order to identify a stable, core set of taxa in the phylogenetic tree that reliably lead to a well-resolved phylogeny. We identified a set of rogue taxa that exhibited long branch attraction behavior (see below). With the increased number of genomes, we identified a stable core tree of cyanobacterial taxa (Fig 2). Bayesian analyses show that branching relationships in analyses with core taxa have 100% posterior probabilities, although these support values must be interpreted with caution as concatenated genomic datasets often converge to a full support for a single topology [57] (see discussion).

Many studies have shown that *Gloeobacter* forms the earliest branch in the cyanobacterial division. The next well resolved lineage to branch off gave rise to the SynPro clade, a group that included taxa such as *Cyanobium*, *Prochlorococcus*, and the small diameter *Synechococcus* (0.6–1.7 *μm*; [58]). The next major branch gave rise to the “LPP group B” clade (including many, but not all, taxa in the Pseudanabaenaceae *sensu* [59]) and includes the taxa *Leptolyngbya*, *Nodosilinea*, and the nitrogen-fixing *Synechococcus* PCC 7335 (which as a slightly larger cell diameter; 1.9–2.1 *μm*; [58]). The next major branch gave rise to the “AcTh” clade (containing *Acaryochloris*, *Thermosynechococcus*, thermophilic *Synechococcus* PCC 6312, and freshwater *Cyanothece* PCC 7425).

As more genome sequences have become available, phylogenetic analyses show the existence of a new well-supported clade, consisting of the taxa *Oscillatoria*, *Trichodesmium*, *Arthrospira*, and *Lyngbya* (making up Oscillatoriales *sensu stricto*; [59]). Most Bayesian analyses showed that this clade is sister to two large clades (previously identified as SPM and PNT). The SPM clade includes marine and freshwater *Cyanothece* (which are polyphyletic), two *Pleurocapsa* species (which are polyphyletic), *Halothece*, *Prochloron*, *Moorea*, and *Coleofasciculus*. The (here renamed) NX clade contains members of the Nostocales (including the Stigonemataceae), and other taxa (*Chamaesiphon*, *Crinalium*, *Chroococcidiopsis*, *Gloeocapsa* (polyphyletic), and *Synechocystis* (polyphyletic)).

**Long branch attraction artifacts in the cyanobacterial tree.** Phylogenetic analyses with all cyanobacterial taxa resulted in a topology different from the core phylogeny, with many long branching taxa branching deep or together (not shown). Analyses with different combinations in taxa often resulted in topologies with varying deep branching relationships. Exhaustive taxon addition and subtraction experiments (S1 Results) showed that several taxa with long branches exhibit long branch attraction artifacts (LBA, [60]), and hence we will refer to them here as “rogue taxa”. Rogue taxa included the Octopus Spring clade (*Synechococcus* PCC 7336, *Synechococcus* JA-2-3B’a(2–13), and *Synechococcus* JA-3-3Ab), *Prochlorothrix hollandica* PCC 9006, *Synechococcus elongatus* PCC 6301 and PCC 7942, the *Pseudanabaena* clade, *Geitlerinema* PCC 7407, *Geitlerinema* PCC 7105, and *Oscillatoriales* cyanobacterium JSC-12. Such rogue taxa branched in different locations depending on the presence of other rogue taxa in the analysis, using both Maximum Parsimony and Bayesian methods. For example, in most analyses the *Pseudanabaena* clade would branch between SynPro and LPP-B. However, in the presence of *Prochlorothrix hollandica* or *Synechococcus elongatus* the *Pseudanabaena* clade would branch deep in the tree (also often resulting in anomalous relationships among the core cyanobacterial tree).

To determine where the most likely branching position of the rogue taxa, analyses were performing using the core taxa plus each rogue taxon individually in order to isolate long branching affects. Next, other rogue taxa were added to the analysis to test for long branch attraction artifacts. Finally, the existence of rare genetic features—an intron in the tRNA-Leu_*UAA*_ gene and an intein in the DnaE1 protein—in the genomes of the rogue taxa were examined (S1 Results). Alignments showed that these rare genetic features had commonality of sequence as well as insertion position (S1 and S2 Figs) and thus were most likely gained once in the evolutionary history of the Cyanobacteria. Some higher clades contained a mixture of taxa that have or lack these genetic features, and the most parsimonious explanation for this distribution pattern is convergent loss of the intron or intein over time.

In the case of the *Pseudanabaena* clade, this group contains both the intron and the intein. Because both of these rare genetic features were likely gained in later-diverging taxa, the presence of these features is inconsistent with *Pseudanabaena* branching deep in the tree. Similarly, some analyses show the *Synechococcus elongatus* clade branching sister to the SynPro clade in the presence of other rogue taxa. However, most analyses show this clade branching higher in the tree, between SynPro and LPP-B. The presence of both the intron and the intein in this clade is inconsistent with this group branching sister to SynPro and is consistent with it branching between SynPro and LPP-B (the latter group having these two rare genetic features). A schematic phylogram showing the consensus branching location of the rogue taxa is shown in Fig 3.

**Time-calibration.** We performed relaxed molecular clock analyses with two alternative constraints on the maximum age of the root node of either 2.7 Ga (consistent with the geologic record) to 3.8 Ga (the end of late heavy bombardment). In all analyses, we estimated a root age that was identical to the maximum age allowed for the root (Table 1, S2 Table). This result was likely artificial, and a reflection of not having any outgroups with age constraints (none are currently available for the bacterial domain of life). Changing the root maximum age from 2.7 Ga to 3.8 Ga resulted in older ages for the basal nodes of the tree (S3 Fig). Nodes in the middle and upper part of the tree, however, were not significantly affected as their ages were more influenced by age constraints in the middle part of the tree. For the remaining analyses, we present results only for trees calibrated to 2.7 Ga in the main text. However, results are qualitatively the same even when the more conservative age estimate of 3.8 Ga is used or with alternative sampling of rogue taxa (S1 Results).

**Table 1:** Ranges of estimated node ages^†^ for major cyanobacterial clades.

Clade	Age Constraints		Range in mean ages	Eon
	Minage	Maxage		
Cyanobacteria	2.320	2.700	2.699^‡^	Archean
SynPro basal node			2.398–2.456	Paleoprot.
SynPro clade			1.256–1.430	Mesoprot.
*Synechococcus-Prochlorococcus*			0.889–1.003	Neoprot.
*Synechococcus*			0.787–0.885	Neoprot.
*Prochlorococcus*			0.677–0.763	Neoprot.
*Cyanobia*			0.868–0.983	Neoprot.
LPP basal node			2.246–2.293	Paleoprot.
LPP			1.910–2.031	Paleoprot.
Ac-Th basal node			2.105–2.219	Paleoprot.
Ac-Th			1.595–2.082	Paleoprot.
Osc-SPM-NX			1.988–2.058	Paleoprot.
Osc			1.869–1.932	Paleoprot.
SPM-NX			1.942–2.000	Paleoprot.
SPM			1.713–1.795	Paleoprot.
NX			1.887–1.933	Paleoprot.
*Nostocales*	1.650	2.320	1.651^‡^	Paleoprot.
*Pleurocapsales*+			0.622–0.689	Neoprot.
Multitrichomous filaments			1.193–1.270	Mesoprot.
False Branching			1.417–1.426	Mesoprot.
Filamentous Sheaths∧	1.900	2.450	1.988–.331	Paleoprot.

^†^ Ages are in Ga, error reported is one standard deviation.

^‡^ Estimated age abuts an age constraint, and therefore the error is near zero Ga, and the range in mean ages is the same.

^+^ The Pleurocapsales are not a monophyletic group; they are defined here as the clade containing *Stanieria, Xenococcus* and *Pleurocapsa* sp. PCC 7319.

∧ Character filamentous sheaths reconstructs to slightly different locations in the tree depending on the unique taxa in each tree.

**Phylogenetic signal.** We tested for the presence of phylogenetic signal in the coded binary discrete traits (defined in Box 1) using Pagel’s *λ* given the phylogenomic trees. We observed substantial phylogenetic signal for nearly all traits studied (p <0.01; Table 2). The only trait lacking significant phylogenetic signal was periphytic growth, while we found only weak significance for microbial mats.

**Table 2:** Epoch model fits and phylogenetic signal.

	Δ lnL†‡	Shift Age (Ga)†^+^	Shift cluster	*Log*_10_ Rate multipliers	Phylogenetic signal (*λ*)†	P value ∧
Trichomes	5.51(3.9–6.3)	1.7(1.7–1.8)	Mesoproterozoic	(-4.0, -3.0)	0.99(0.98–1)	***
Filamentous morphology	4.90(4.2–5.6)	1.7(1.6–1.8)	Mesoproterozoic	(-2.2, -2.3)	1	***
Uniseriate trichomes	4.65(2.8–5.0)	1.7(1.7–1.8)	Mesoproterozoic	(-2.2, -1.8)	1	***
Heterocysts	4.03(3.7–4.2)	1.7(1.7–1.8)	Mesoproterozoic	(-2.8, -4.5)	0.8(0.79–0.8)	***
Hormogonia	4.00(3.6–4.5)	1.7(1.7–1.8)	Mesoproterozoic	(-2.6, -2.9)	0.96(0.96–0.97)	***
Motility	3.46(2.6–3.6)	1.7(0.4–1.8)	Mesoproterozoic	(-3.1, -2.3)	0.98(0.97–0.98)	***
Fission in multiple planes	3.17(2.8–3.6)	1.5(1.4–1.5)	Mesoproterozoic	(-1.7, -4.0)	1	***
False branching	1.42(1.3–1.6)	1.5(1.5–1.5)	Mesoproterozoic	(-1.5, -1.7)	1	**
True branching	1.51(1.4–1.6)	1.1(1.1–1.3)		(-1.3, -3.0)	1	**
Baeocytes	2.76(2.0–2.8)	1.0(0.9–1.0)	Neoproterozoic	(-4.0, -3.5)	1	**
Akinetes	2.60(2.2–2.7)	1.0(1.0–1.0)	Neoproterozoic	(-4.0, -4.1)	1	***
Freshwater habitat	3.96(3.7–4.8)	0.8(0.8–0.9)	Neoproterozoic	(-2.5, -1.8)	1	***
Thermophilic	2.43(2.1–2.8)	0.8(0.8–0.9)	Neoproterozoic	(-1.5, -4.0)	0.94(0.92–0.96)	**
Epi/Endolithic	1.35(1.3–1.5)	0.8(0.8–1.2)	Neoproterozoic	(-0.7, -1.1)	1	**
Marine planktonic	1.47(1.2–1.5)	0.7(0.7–0.7)	Neoproterozoic	(-2.6, 3.0)	0.87	***
Gas vesicles	2.03(1.9–2.1)	0.6(0.6–1.5)	Neoproterozoic	(3.0, -2.7)	1	**
Extracellular sheath	3.24(3.1–3.4)	0.5(0.5–0.5)		(0.8, 2.0)	0.74(0.72–0.75)	**
Freeliving	2.41(2.3–2.5)	0.5(0.5–0.5)		(2.0, 1.0)	0.91(0.9–0.93)	***
Growth habit	2.10(1.9–2.3)	0.5(0.5–0.5)		(1.0, 2.0)	0.82(0.8–0.82)	***
Epiphytic	1.12(1.0–1.2)	0.5(0.5–0.5)		(-3.3, -0.2)	1	**
Periphytic	0.90(0.9–1.0)	0.4(0.2–1.3)		(1.3, 2.4)	0.94(0.44–1)	.
Microbial mats	3.18(2.8–3.6)	0.1(0.1–0.1)		(3.0, 0.7)	0.67(0.61–0.73)	*
Cell diameter	2.85(2.8–3.0)	0.1(0.1–0.2)		(1.6, -2.0)	0.77(0.76–0.78)	***
Nitrogen fixation	1.95(1.8–2.0)	0.1(0.1–0.1)		(3.0, -0.8)	0.88(0.88–0.89)	***
Mucilage	1.25(1.2–1.4)	0.1(0.1–0.1)		(0.7, 2.1)	0.88(0.87–0.89)	***

^†^ Range in parentheses indicates range of estimates across 20 trees from 2 posteriors

^‡^ Difference between best-fit epoch model and a constant rate model

^+^ Age indicates the best estimate of the timing of a shift under an epoch model.

∧. p <0.1;

* p <0.05

** p <0.01;

*** p <0.001

**Fig 4:**
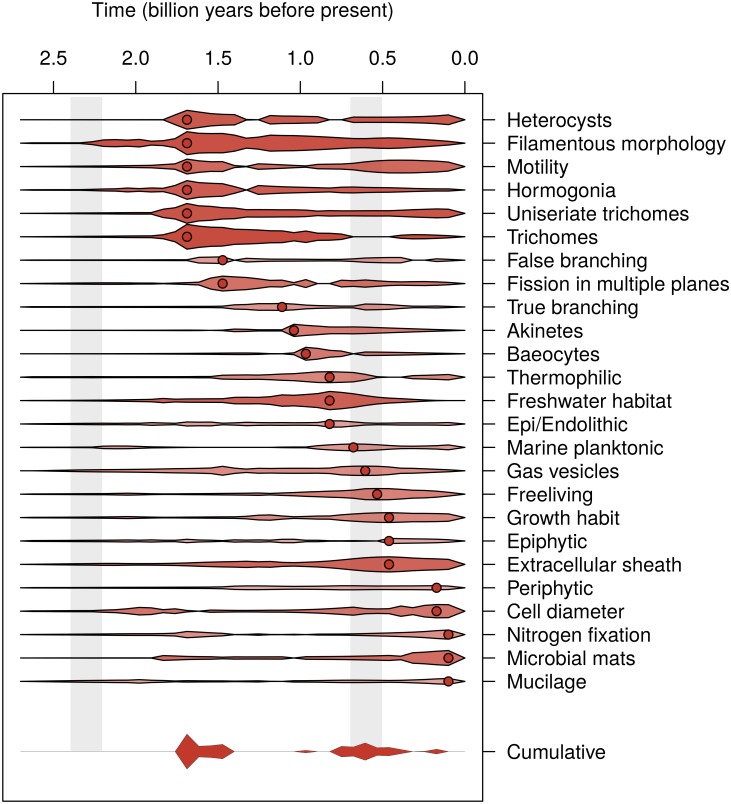
Location of Support for a Shift for Each of 25 Phenotypic Traits. The width and color intensity of each violin plot is proportional to the increase in support (likelihood units) for the epoch-model over the constant rate model (taken as the median for each time point over 2 sets of 10 trees taken from the posterior distribution of two different taxa sets). Points indicate the location of the maximum likelihood estimate for the location of the time-shift. Gray bars indicate the location of the Great and Neoproterozoic Oxidation Events. On the far right, the cumulative likelihood support is depicted (with all values <6 likelihood units less than the maximum set to 0). Two sets of major peaks can be found at 1.7 Ga and 0.6 Ga.

**Discrete trait epoch model and bursts of evolutionary innovation in the Cyanobacteria.** In addition to finding substantial phylogenetic signal for nearly all binary traits (see above; Table 2), we also found strong statistical support for the epoch model across traits. A joint model with a single rate shift and single rate multiplier across traits finds a (statistically significant) average Δ*AIC* of 7.34 calculated over 20 trees sampled from the posterior probability distribution, indicating strong evidence across the complete dataset of a shift in evolutionary rates. The average shift location is at 1.24 Ga and corresponds overall to an approximately 5-fold reduction in phenotypic rates. In other words, we find strong evidence for rapid gains or losses in morphological and ecological character states early in the Earth’s history. Examination of likelihood profiles across different time shift locations suggests that support for the epoch-model varies among traits, with some traits favoring an early rate-shift in the Mesoproterozoic, and other traits favoring a later rate-shift in the Neoproterozoic (Fig 4 and S4 Fig; Table 2). After fitting independent epoch models to each trait across all possible time-shift locations, we found a maximum peak centered around 1.7 Ga and a secondary peak around 0.6 Ga that differed by only 1.20 AIC (Fig 4).

The presence or absence of trichomes was the only trait that supported the epoch model over the single-rate model with Δ*AIC* > 4. Heterocysts, filamentous morphology, uniseriate trichomes, and hormogonia had Δ*AIC* > 3 over the single-rate model (Table 2, S4 Fig). All traits associated with the Mesoproterozoic rate shift universally demonstrated a decrease in the transition rate of both gains and losses (Table 2, S4 Fig). This indicates that there is evidence for an overall slowing of discrete character evolution across traits during this time period, which could reflect fixation in ecological and physiological character states after an initial burst of character state changes.

Traits associated with the Neoproterozoic rate shift demonstrated more varied dynamics, often with major rate changes in either the rate of gains or losses of a particular character state (Fig 4 and S4 Fig). The peak between 0.8–0.5 Ga near the end of the Neoproterozoic appears associated with a suite of traits related to invasion of marine planktonic habitats, including increased rates of evolution of extracellular sheaths, freeliving/planktonic life history, gas vesicles, and invasion of saline habitats. We also found a decrease in the rate of transitions to and from thermophilic character states, as well as a secondary peak in the evolution of motility during this time (Table 2, S4 Fig).

A few traits exhibited moderate support for Neoproterozoic rate shifts (akinetes, true branching and baeocytes), as well as some traits supporting more recent (Phanerozoic) shifts toward mostly higher rates of evolution (cell diameter, presence in microbial mats, and mucilage). Finally, several traits show bimodal support, with peaks both in the Mesoproterozoic and either the Neoproterozoic or Phanerozoic (e.g. nitrogen fixation, motility, and gas vesicles, Table 2).

**Simulation study of character evolution along the cyanobacterial phylogeny.** Simulation results indicate that the concentration of support for shifts around 1.8–1.6 and 0.8–0.6 Ga may be affected by the overall structure of the tree. In particular, simulations under a constant rate model tend to, on average, show the strongest support for a shift at 0.6 Ga (S5 Fig). These results indicate that the particular patterns we found for the epoch model can, under some circumstances, result in artifactual support for a shift even when the data are simulated under a constant-rate model where none has truly occurred. Across simulations, we show no evidence of a bias to detect a shift in the Paleo- or Meso-proterozoic, although some individual simulations do detect a peak in this region, even under the constant-rate model.

However, it is notable that in our empirical data, all the traits that display a shift older than around 1.0 Ga also demonstrate decelerations of evolutionary rate. By contrast, our simulations under a constant-rate model suggest an overall bias toward estimating an increase in rates at the 1.6–1.8 Ga boundary (71.8% of simulations), rather than a decrease (S6 Fig), when fit to the epoch model. In fact, examination of the parameter estimates across the empirical vs. simulated datasets shows opposing trends through time. While under a constant rate model, our simulations suggest that it is more common to estimate accelerating trait evolution for shifts older than 1 Ga, and decelerating evolution for shifts younger than 1 Ga. This is precisely the opposite of the pattern we observed (S6 Fig). Though the exact location of the shift at 1.6–1.8 Ga can be influenced by the shape of the phylogeny, there is an opposing bias against finding decelerating rates overall. Thus, the fact that so many of the traits we examine demonstrate a strong overall signal of decelerating rates with high support for a shift model supports the conclusion that a concerted, multi-trait rate shift did in fact occur early in the history of the Cyanobacteria. We find little evidence that topological or branch length errors will affect estimation of rate shifts. Rather, only the transition rate parameters have a significant impact on the relative location or the magnitude of shifts recovered under the epoch model (see S1 Results).

**Ancestral state reconstruction of traits found in the fossil record.** We performed ancestral state reconstruction on these time-calibrated phylogenies using parsimony and maximum likelihood under a constant-rate model. We also analyzed the data under an epoch model when the epoch model was suggested over the constant-rate model by model selection. Since the epoch model is a simple extension of the standard maximum likelihood methods for ASR, we can use model testing to determine whether or not there is evidence for rate heterogeneity in the evolution of a particular trait. In most cases, epoch models do not dramatically affect inference of ancestral states (or are not strongly supported over constant-rate models). However, when these models do affect such inferences, we report them below.

**Fig 5:**
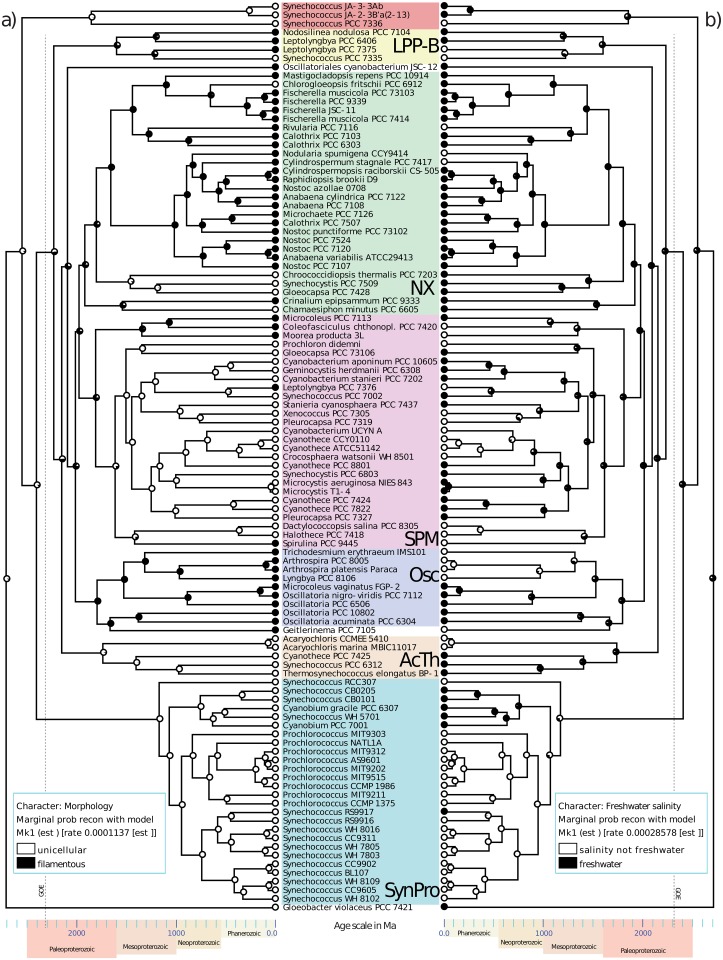
Ancestral State Reconstruction of Morphology and Freshwater Habitat Salinity. Reconstruction of binary traits for morphology (a) and freshwater habitat salinity (b), where the node color indicates the probability of the inferred character state. Branch lengths in the trees are proportional to time and the geologic age scale is shown at the bottom (with ages in Ma).

Ancestral state reconstructions allowed us to infer the ancestral characteristics of important nodes in the cyanobacterial phylogeny—providing a rough guide for placing fossil taxa in the context of extant diversity (Table 3). First, we can consider a number of morphological traits relating to filamentous morphologies, many of which are preserved in the fossil record. Under a constant-rate model, ASR showed that the earliest nodes of the tree were likely unicellular (Fig 5). We show that the epoch-model is favored over a constant-rate model, and suggests multiple origins filamentous growth forms prior to 1.6 Ga, while the constant-rate model suggests that most filamentous morphologies originated around 2.0–2.1 Ga in the freshwater ancestor of the Osc-SPM-NX clade (Fig 5; Tables 1–3). Both models, however, reconstruct multiple origins of filamentous growth, with no origins younger than 1.5 Ga.

**Table 3:** Inferred node ages and associated morphological and ecological traits^†^ .

Age	Node Age (Ga)	Key traits at node	Node identity and location	Inferred habitat at node
Paleoprot.	pre-GOE (pre-2.32)	< 2.5 μm, unicellular binary fission benthic/sessile epi/endolithic (?) extracellular sheath (?) absence of motility	cyanobacterial clade SynPro basal node	freshwater
Paleoprot.	2.2–2.3	extracellular sheath epi/endolithic	LPP-B basal node	freshwater
Paleoprot.	2.0–2.1	> 3.5 μm, filamentous isopolar filaments (?)	Osc-SPM-NX clade	freshwater
Paleoprot.	1.9–2.0	isopolar filaments (?) uniseriate trichomes (?)	LPP-B ancestor	freshwater
Paleoprot.	1.9	uniseriate trichomes hormogonia	Oscillatoriales clade	freshwater
Paleoprot.	1.7	fission in multiple planes	within NX (Chroococcidiopsis- Gloeocapsa -Synechocystis)	freshwater
Mesoprot.	<1.65^‡^	akinetes, heterocysts nitrogen fixation uniseriate trichome motility/gliding motility hormogonia isopolar filaments (?)	Nostocales	freshwater
Mesoprot.	1.5	motility/gliding motility hormogonia isopolar filaments	within SPM (Coleofasciculus—Microcoleus—Moorea)	marine(?)
Mesoprot.	1.5	motility/gliding motility	within Osc Arthrospira—Lyngbya—Trichodesmium)	marine(?)
Mesoprot.	1.5	planktonic	within LPP-B (Leptolyngbya—Synechococcus)	marine(?)
Mesoprot.	1.4	false branching filamentous heteropolar filaments fission in multiple planes	within Nostocales (Calothrix-Rivularia) within Nostocales (Chlorogloeopsis—Fischerella—Mastigocladopsis)	freshwater
Mesoprot.	1.2–1.4	planktonic	SynPro clade	marine(?)
Mesoprot.	1.3	multitrichomous filaments	within Osc (Coleofasciculus—Microcoleus)	marine(?)
Mesoprot.	1.1	> 2.5 μm, benthic/sessile binary fission	Within SPM (Dactylococcopsis—Halothece—Spirulina)	marine
Neoprot.	0.9–1.0	planktonic	within SynPro (Synechococcus—Prochlorococcus)	marine
Neoprot.	0.9	multiseriate trichome	within Nostocales (Chlorogloeopsis—Fischerella)	freshwater
Neoprot.	0.9	planktonic	Within SPM (Cyanobacterium—Geminocystis—Leptolyngbya—Stanieria—Synechococcus)	freshwater
Neoprot.	0.8	planktonic	Within SPM (marine and freshwater Cyanothece spp.—Crocosphaera—Microcystis—Pleurocapsa—Synechocystis)	freshwater
Neoprot.	0.7	nitrogen fixation	within SPM (Crocosphaera—Cyanothece) within SPM (Cyanothece—Pleurocapsa)	freshwater
Neoprot.	0.6	fission in multiple planes	within SPM (Pleurocapsa—Stanieria—Xenococcus)	freshwater
Neoprot.	0.6	heteropolar filaments	within Nostocales (Calothrix—Microchaete)	freshwater
Phanerozoic	0.4	true branching false branching	within Nostocales (Fischerella)	freshwater
Phanerozoic	0.3	planktonic nitrogen fixation	within SPM (marine Cyanothece spp.—Crocosphaera)	marine

^†^ Node ages and inferred ancestral states calculated using ASR, summarizing the major evolutionary trends observed. Nodes are listed when the appearance of novel traits are inferred in the node using ASR. The trait that appears is listed, along with the corresponding node. Only nodes older than 0.4 Ga are shown. Nodes labeled as (possibly) marine indicates that there is uncertainty in the ancestral state reconstruction of this node, so the node could be freshwater or marine.

^‡^ Inferred age is against the minimum age constraint allowed for this group—thus it is possible that this node and associated traits are younger than 1.65 Ga.

We document ancestral state reconstructions that infer times of trait evolution that conflict with previous estimates. As a primary example, consider cyanobacterial filaments that have more than one trichome per filament (these are called multitrichomous). Molecular age estimates of multitrichomous filaments were not consistent with fossil age constraints. Multitrichomous morphologies are found in several recognized cultured genera including *Microcoleus* [61], *Symplocastrum* [62], *Coleofasciculus* [63], and *Kastovskya* [64]. ASR (S8 Fig) showed multitrichomous filaments originating independently in two parts of the tree—in *Coleofasciculus—Microcoleus* PCC 7113 in the SPM clade (∼1.3 Ga, possibly in marine environments) and in *Microcoleus vaginatus* in the Oscillatoriales. In the microfossil record, multitrichomous morphologies are interpreted to have first appeared in the Gaoyuzhuang Formation, about 1.56 Ga in the Mesoproterozoic [65–67]. Thus, the molecular age for the first appearance of multitrichomous morphologies was significantly younger than those in the microfossil record. Since there were only a few multitrichomous taxa in the phylogenomic tree, more genome sequences will be needed to obtain a better age estimate of this particular morphological trait.

Molecular age estimates of akinetes were also found to not be consistent with fossil age constraints. In all analyses, the estimated age was the minimum age allowed for this group (1.65 Ga; Table 1). In the fossil record, akinetes are often reported in Mesoproterozoic rocks dating between 1.65 and 1.03 Ga (Riphean 1 and 2; [68, 69]). The molecular age, however, was not consistent with the earliest age of akinete appearance (2.1 Ga) proposed by [44]. Thus, the molecular age for the first appearance of akinetes was largely consistent with the microfossil record, although not with the earliest interpretation of akinetes in the fossil record.

By contrast, molecular age estimates of false branching filaments were found to be generally consistent with fossil age constraints. In the fossil record, falsely branched filamentous morphologies are only sparsely reported, described as *Palaeocalothrix*. *Palaeocalothrix*-like morphologies are reported in the Gaoyuzhuang Formation (1.56 Ga; [70]) and in the Society Cliffs Formation, Bylot Supergroup (1.2 Ga; [71, 72]). Molecular age estimates of cell diameter were also consistent with the fossil record. The evolution of cell diameter (S13 Fig) shows that the earliest nodes in the Cyanobacteria were most certainly less than 2.5 *μm* in diameter. A clear pattern of cell diameters becoming larger than 2.5 *μm* can be traced back to the ancestor of the Osc-SPM-NX clade, which has a predicted age of ∼2.0–2.1 Ga (Table 1). The phylogenetic pattern for cell diameters greater than 3.5 *μm* is nearly identical to that for 2.5 *μm* (S13 Fig). Cell diameters much larger than 3.5 *μm* are acquired independently within the Oscillatoriales, SPM, and NX clades, high up in the tree (not shown).

Finally, we also found many examples of multiple independent origins of traits among distantly related lineages of Cyanobacteria. Consider the pattern of cell division in Cyanobacteria, which can be preserved in the fossil record because this often influences overall morphology. Most Cyanobacteria divide using binary fission, where the plane of division is in one plane. Binary fission was most likely the ancestral state (S8 Fig). In contrast, fission in more than one plane of division (sometimes called multiple fission) is found in multiple clades, high up in the tree (S8 Fig). In the NX clade, fission in multiple planes originated in the ancestor to *Chroococcidiopsis—Gloeocapsa* PCC 7428—*Synechocystis* PCC 7509 (∼1.7 Ga), and in the ancestor to *Chlorogloeopsis—Fischerella—Mastigocladopsis* (∼1.4 Ga), both likely originating in freshwater environments (Table 3). This division pattern also may originated at least five times independently in the SPM clade (including the freshwater Pleurocapsalean taxa *Stanieria—Xenococcus—Pleurocapsa*, ∼0.6 Ga).

Another example of repeated convergent evolution is seen in lineages with uniseriate trichomes, which appeared in three major clades originating independently. Epoch models suggest that rates of evolution of this trait were higher prior to the Mesoproterozoic, as there are likely mutiple origins of uniseriate trichomes originating in the freshwater ancestors of the LPP-B clade (∼1.9–2.0 Ga), the Oscillatoriales (∼1.9 Ga), and the Nostocales (<1.65 Ga; S9 Fig, Table 3). There are also multiple scattered origins of uniseriate trichomes high up in the SPM clade. Multiseriate trichomes (Box 1, S9 Fig) likely originated high up in the cyanobacterial tree, in at least four clades. This morphology originated in the freshwater ancestor to *Chlorogloeopsis-Fischerella* (∼0.9 Ga), and high up in the LPP-B clade and multiple times high up in the SPM clade.

We also find multiple origins of branching. In the Cyanobacteria, the branching of filaments manifests as two main types—called true and false branching (Box 1). ASR showed that true branching is inferred to have appeared several times in freshwater taxa—in *Fischerella* spp. ∼0.4 Ga and in *Mastigocladus* (S10 Fig, Table 3). Two later origins of true branching occurred high up in the LPP-B and SPM clades. False branching also appeared to have originated several times independently. For example, constant-rate ASR (S10 Fig) suggests that false branching appeared twice in the tree, first in the freshwater ancestor to *Rivularia* and *Calothrix* sp. PCC 7103 and PCC 6303 (∼1.4 Ga) and then in the freshwater ancestor to *Microchaete* and *Calothrix* sp. PCC 7507(∼0.4 Ga; *Calothrix* is not a monophyletic genus).

A further example of convergent evolution is seen in hormogonia (Box 1), which are often accompanied by traits such as gliding motility or gas vesicles that enhance dispersibility [73]. Hormogonia appeared to have originated in several clades independently, with a phylogenetic distribution pattern that is similar to filamentous morphologies (S11 Fig). ASR (S11 Fig) inferred the presence of hormogonia in the freshwater ancestors of the Oscillatoriales sensu stricto (∼1.9 Ga), the freshwater ancestor to the Nostocales (< 1.65 Ga) and within the SPM clade in the (possibly marine) ancestor to *Coleofasciculus-Microcoleus-Moorea* (∼1.5 Ga). However, relatively high support for an epoch model suggests that rates of transitions between states may have been higher prior to 1.7 Ga, making inference as to the exact number of origins more difficult. Molecular age estimates for the first appearance of hormogonia (and also sheathed filaments) was consistent with the microfossil record. In the fossil record, hormogonial microfossils have been described as those morphologies that resemble those found in the Oscillatoriales and Nostocales [74]. First reports of “hormogonian Cyanobacteria” are from the Belcher microbiota, dated at 1.9–2.0 Ga, although their distribution is reported to be sparse [75]. Later deposits (Lower and Middle Riphean, 1.2–1.65 Ga) regularly describe morphologies of sheaths thought to derive from hormogonian Cyanobacteria [42].

We also see convergence in filamentous morphologies, which can be further distinguished on the basis of the polarity of filaments (isopolar versus heteropolar; Box 1, S12 Fig). Isopolar filaments are more phylogenetically widespread among clades compared to heteropolar filaments. Both isopolar and heteropolar filamentous morphologies likely originated several times independently in the tree. Isopolar filaments may have appeared in the freshwater ancestor of the LPP-B (1.9–2.0 Ga) and in the freshwater ancestor of the Osc-SPM-NX clade (2.0–2.1 Ga). They also likely appeared multiple times independently in the SPM (including the possibly marine ancestor of *Coleofasciculus—Microcoleus—Moorea* ∼1.5 Ga). Heteropolar filaments, in contrast, likely originated at least twice high up in the SPM, and twice in the Nostocales (in the freshwater ancestor of *Calothrix—Microchaete* ∼ 0.6 Ga, and in the freshwater ancestor to *Calothrix—Rivularia* ∼1.4 Ga).

By contrast, some traits show evidence of single origins. For example, ASR under all models showed a single origin of heterocysts were likely present in the ancestor to the Nostocales (including the Stigonemataceae; (S7 Fig). We also show that akinetes originated in two clades in the Nostocales (S7 Fig). However, since this is a complex trait (see methods section), it is more likely that this trait is homologous and originated once in the ancestor to the Nostocales, but was subsequently been lost in some taxa (or not observed or reported in cultured representatives).

**Ancestral state reconstruction of traits not found in the fossil record.** Many cyanobacterial traits are not explicitly found in the fossil record, therefore ASR can provide fundamental insights into the evolutionary history of morphologies and ecologies, independent of the fossil record.

ASR showed that the earliest nodes of the tree were likely freshwater (low salinity, not marine; Fig 5). ASR predicted that marine salinity did not evolve until much higher up in the tree. Marine (also planktonic) Cyanobacteria may have first appeared in the ancestor to the SynPro clade, ∼ 1.2–1.4 Ga, and possibly in the (also having gliding motility, hormogonia, isopolar filaments) ancestor to *Arthrospira—Lyngbya—Trichodesmium* ∼1.5 Ga (Table 3). Marine planktonic growth was certainly present in the ancestor to *Synechococcus—Prochlorococcus* (∼ 0.9–1.0 Ga). SPM with higher salinity preferences are also predicted to have originated ∼1.1 Ga in the (> 2.5 μm, benthic/sessile, dividing by binary fission) ancestor to *Dactylococcopsis—Halothece—Spirulina*. Later on, Phanerozoic origins of marine taxa are predicted to have appeared in the planktonic nitrogen-fixing *Crocosphaera—Cyanothece* and the benthic/sessile *Pleurocapsa—Xenococcus*.

ASR also predicted that the earliest nodes in the tree had a benthic or sessile growth habit (not planktonic; S3 Fig). Planktonic growth appeared to have originated later in the tree, first in the possibly marine ancestors of the SyPro clade (1.2–1.4 Ga) and *Leptolyngbya—Synechococcus* in the LPP-B clade (∼1.5 Ga). Much later appearances of planktonic growth were inferred to have occurred in the SPM clade: in the ancestor of *Cyanobacterium—Geminocystis—Leptolyngbya—Stanieria—Synechococcus* (∼0.9 Ga) and in the ancestor of *Crocosphaera—Cyanothece—Microcystis—Pleurocapsa—Synechocystis* (∼0.8 Ga). After the latter group became planktonic, they appear to have gained the ability to fix nitrogen (∼0.7 Ga) and then colonize marine environments (∼0.3 Ga; Table 3).

Interestingly, ASR predicted that the early nodes in the tree were epilithic or endolithic (i.e. had an association with rocky substrates) and may have had an extracellular sheath (S14 Fig). This correlates with them being benthic or sessile and not planktonic. Also, the phylogenetic distribution of extracellular sheath had a similar pattern as filamentous morphology and hormogonia (Fig 5 and S11 Fig). Epoch models showed moderate support for increases in the rate of gains and losses of an extracellular sheath near the end of the Neoproterozoic.

Nitrogen fixation is widely distributed in the Cyanobacteria, and likely was not ancestral but arising in multiple clades independently S15 Fig. It appeared to have originated (somewhere) in the LPP-B clade (after 1.9–2.0 Ga), somewhere in the Osc clade (after 1.9 Ga), in the ancestor to the Nostocales (< 1.65 Ga in freshwater environments). In the SPM clade, nitrogen fixation was gained in the freshwater ancestor of *Cyanothece—Pleurocapsa* and the freshwater ancestor of *Crocosphaera—Cyanothece* (both ∼ 0.7 Ga; Table 3).

Cyanobacterial presence in thick, laminated microbial mats was a trait that was distributed widely and sparsely in the tree (S15 Fig). Presence in microbial mats originated in the Octopus Spring clade, within the Oscillatoriales, several times high up in the SPM clade and several times high up in the NX clade. Although ASR suggested this trait arose multiple times, statistical tests showed that there was little phylogenetic signal in the microbial mat trait, and epoch models suggest a recent increase in the rate of gains the trait only at the very tips of the phylogeny (Table 2). An interesting note, however, is that the phylogenetic distribution of nitrogen fixation and presence in microbial mats in extant taxa was quite disjoint (S15 Fig). Also, the distribution of extracellular sheath was much more widespread in the tree than growth in microbial mats, the latter of which appeared to have originated much later (S14 and S15 Figs). Most Cyanobacteria are non-motile, and lack of motility was most likely an ancestral trait, arising before the GOE (S16 Fig). We detect higher rates of evolution in motility prior to 1.5 Ga, with motility likely first appearing in the ancestor of the Nostocales (<1.65 Ga), in the ancestor of *Coleofasciculus—Microcoleus—Moorea* (∼1.5 Ga) and *Arthrospira—Lyngbya—Trichodesmium* (both possibly marine, and both ∼1.5 Ga). Gliding motility (the ability to move across a solid surface) was found in many of the taxa and clades also exhibiting motility, and exhibiting epi/endolithic growth (S14 Fig). In contrast, motility through the presence of gas vesicles was sparsely distributed, arising multiple times high up in the tree (S11 Fig). Gas vesicles likely appeared high up in the LPP-B and Oscillatoriales, and high up in the SPM and NX clades, and is not predicted to have originated until the Phanerozoic.

## Discussion

**Core cyanobacterial tree.** As more cyanobacterial genomes are released, the evolutionary relationships within and between major clades become better resolved and a much clearer picture of evolutionary history emerges. Seven major clades (Fig 2) are well-resolved and exhibited stable phylogenetic relationships, outlining the well-resolved core of the cyanobacterial tree. Many of these clades emerged in previous studies when only 16S rRNA [76] or a small number of genomes were available [18].

We show clear delineation of a new clade (previously identified as OSC in [76]) that consists of the taxa *Oscillatoria, Microcoleus vaginatus, Trichodesmium, Arthrospira,* and *Lyngbya*. Most Bayesian analyses revealed that this clade most likely branches basal to two large clades (SPM and NX, previously identified as the PNT clade). Some of the taxa in this clade are included in Oscillatoriales [77], a clade defined using morphological features. All of the taxa in this clade are included in the Oscillatoriales *sensu stricto* [59].

As more strains are sequenced, it becomes ever more clear that there is a need for a major revision of cyanobacterial taxonomy. As has been noted by others, many species with genome sequences are polyphyletic. These include *Anabaena, Calothrix, Cyanothece, Gloeocapsa, Leptolyngbya, Microcoleus, Nostoc, Pleurocapsa, Synechococcus*, and *Synechocystis*. Second, an increasing number of studies have shown that conventional taxonomic classification based on morphology does not agree with phylogenetic relationships [78, 79]. Indeed, nearly all conventionally delimited subdivisions (Chroococcales, Pleurocapsales, Oscillatoriales, Nostocales) are non-monophyletic. The current selection of sequenced taxa (Fig 2) show that Stigonematales are monophyletic, however an analysis of 16S with a larger set of taxa show this group is also non-monophyletic [80]. As more sequences become available and phylogenetic patterns reach a consensus, cyanobacterial taxonomy should be able to transition toward a more robust phylogenomic taxonomic system that is an extension of the phylogenetic species concept [81, 82].

Previous research has shown concatenation can result in inflated posterior probabilities even though individual gene trees are in conflict [57, 83]. However, we note that our resulting topology reflects an emerging view of cyanobacterial phylogenetics that is concordant with single-gene topologies from previous studies [18, 76]. Extensive congruence tests have been performed on the concatenated proteins in the dataset, using a smaller number of taxa [18, 19]. These tests showed that most of the sequences in the dataset supported the core cyanobacterial tree, and that inclusion of incongruent sequences did not significantly change the topology of the tree. Therefore we conclude that while our posterior probability estimates may be inflated, we view the core phylogenetic relationships of the cyanobacteria as well-supported both by individual genes as well as concatenated gene tree analysis.

**Long branch attraction artifacts in the cyanobacterial tree.** Having a correct tree topology is critical for accurately reconstructing the ancestral states in the tree and for accurately interpreting the early evolution of traits. We show that long branch attraction between a few rogue lineages can radically alter the backbone topology of the tree, producing branching artifacts. Taxon addition and subtraction experiments clearly show how the presence and absence of these rogue lineages affect the topology of the tree, and that alternative topologies can be ruled out by the presence or absence of rare genetic features (such as the intron in the tRNA-Leu_*UAA*_ gene and inteins in the DnaE1 protein).

Multiple studies, for example, showed the *Pseudanabaena* clade as a deeply branching clade [37, 76, 84–86]. We show that this branching location is most likely a long branch attraction artifact and that the *Pseudanabaena* clade likely branches higher up in the tree, between SynPro and LPP-B (Fig 3). This latter branching location is consistent with the presence of an intron in tRNA-Leu_*UAA*_ and inteins in DnaE1, both of which are found in the LPP-B clade.

Similarly, multiple studies show the *Synechococcus elongatus* clade branching sister to SynPro [84–89]. We show that this branching location is also likely a long branch attraction artifact and that *Synechococcus elongatus* most likely branches between SynPro and LPP. This latter branching location is consistent with the presence of both rare genetic features, and it also is consistent with clustering based on operon structure [90].

Many novel lineages appear to branch between the SynPro and LPP-B clades, and their relative branching positions are at present difficult to resolve. It is possible that these lineages are all part of a rapid radiation of lineages that appeared in a relatively short period of time. The sequencing of additional genomes that help to break up these naked, long branches should improve our ability to resolve these phylogenetic relationships in the future.

**Phylogenetic signal.** Statistical tests showed there was strong, statistically significant phylogenetic signal in nearly all the traits analyzed in this study (24 out of 25 traits tested; Table 2). Therefore, we contend that phylogenetic comparative methods (such as ASR) can be used to study the evolution of phenotypic traits in the Cyanobacteria, even if those traits have been gained by lateral gene transfer. Indeed, given statistically significant phylogenetic signal over deep time, this suggests that the rate of lateral transfer is relatively rare in the cyanobacterial phylogeny, at least for the genes underlying the phenotypic traits tested here.

A growing number of genomic analyses are showing that specific parts of the genome experience relatively rapid lateral gene transfer. The parts of the genome involved with these transfer events appear to be sections of the genome associated with mobile genetic elements (transposases, phage genes) [91]. The parts associated with phenotypic and ecological traits, however, do not appear to be rapidly transferred, given the taxa and traits in this study.

**Bursts of evolutionary innovation in the Cyanobacteria.** Considering traits jointly suggests that across the traits we examined, there has been an overall slowdown in the rate of trait evolution across the cyanobacterial phylogeny. Simulation results demonstrate that this is not expected under a constant-rate model, and the fact that all 14 traits we examined that have a Mesoproterozoic rate shift all show decelerations is in opposition to the bias generally found in the simulations (S6 Fig). Furthermore, the joint model is by itself significantly different from the null model under AIC. This strongly supports that the effect we observe is not an artifact of the phylogeny or error, but rather indicative of a general slowdown in rates of character evolution.

Nevertheless, the exact timing of the rate shifts estimated using our method may be influenced by the shape of the phylogeny. While our sampling of taxa is not random and therefore does not represent fully the diversification dynamics of the clade, it is likely that it is reflects some of true dynamics of diversification. The phylogeny has numerous short branches prior to 1.8 Ga, when most of the major clades emerge, as well as an additional diversification of clades (particularly the SPM and SYN-PRO clades) in the Neoproterozoic. While on average, support for these rate shifts in our simulations are much less than what we observe in our dataset, we are nevertheless cautious to conclude that our results reflect the precise timing of rate shifts. Rather, our simulation results suggest that the shape of the phylogeny may concentrate the appearance of rate shifts at these diversification events.

Even so, it is tempting to associate the overall slowdown we observe at the beginning of the Mesoproterozoic, with an overall decrease in rates following a major adaptive radiation of cyanobacterial forms that occurred following the GOE. Traits associated with this rate shift event include major ecological traits such as filamentous morphology, heterocysts, and motility. It appears that these traits rapidly diversified prior to the emergence of the major cyanobacterial clades, and evolved under relative stasis thereafter. Furthermore, regardless of whether we test our results against a tree with the a maximum root age constraint of 2.7 of 3.8 Ga, we observe the same patterns and major timings of events (S1 Table). This indicates that the a model of decelerating rates cannot be rejected by simply increasing the age of the root to a very conservative age (3.8 Ga).

We also observe a series of rate shifts that occur between 0.8–0.5 Ga in the Neoproterozoic. These shifts coincide with the secondary oxygenation event, and are primarily driven by traits associated with invasion of free-living, marine environments by cyanobacterial lineages. As our simulation results suggest that it is common to find elevated support for a shift in this region of the phylogeny, we interpret the finding of a rate shift here with considerable caution. Nevertheless, if real, the particular traits and transitions we identify fit well with a model in which cyanobacterial lineages invade and diversify marine planktonic habitats in conjunction with the Neoproterozoic oxygenation event [92].

**Timing of Traits Found in the Fossil Record.** We lack the necessary outgroups to provide any new insight into the timing of clade origination in Cyanobacteria. Other bacteria were not included in this analysis because there is no general consensus on the deep branching relationships between bacterial divisions (phyla). Secondly, there are little or no available age constraints for the vast majority of non-cyanobacterial taxonomic groups, and therefore no age constraints outside of Cyanobacteria that (at this time) could lead to insights into the age of Cyanobacteria and oxygenic photosynthesis.

In this study, we used two maximum age constraints for the age of Cyanobacteria: 2.7 Ga (which coincides with most of the geochemical trace oxygen) and 3.8Ga (the end of late-heavy bombardment, as a conservative open constraint). We showed that while changing this age resulted in shifts in the age estimates of the basal nodes, they largely did not affect internal node ages whose ages were more highly influenced by additional local age constraints within the clade.

Interpretation of ancient microfossils that are billions of years old is profoundly difficult due to the extreme age of the samples as well as the potential for pseudofossils or dubiofossils and post-depositional microbial contamination [93]. Hence, identifying the first age of appearance of particular morphological traits attributable to Cyanobacteria can be problematic. ASR can help to focus searches for particular morphotypes at different geologic times, and it could help set up a framework for the interpretation of potential fossil candidates as well as the evaluation of established microfossils.

ASR agrees with the age of first appearance of false branching, sheathed filaments, hormogonia, and large cell diameters (attributed to Cyanobacteria) in the fossil record. ASR does not agree with the presence of akinetes (or Nostocales) at 2.1 Ga [44], although it does agree with their younger presence in the Mesoproterozoic (when reports of these microfossils become more frequent; [68, 69]). ASR also does not agree with the age of the first appearance of multitrichomous fossils, however only a few genome sequences with this trait were included in this study so this will need to be confirmed once additional taxa in this group are sequenced.

**Timing of traits not found in the fossil record.** Results in the reconstruction of early trait evolution in Cyanobacteria agree with previous studies performed with fewer genome sequences or with much smaller genetic datasets [18, 19, 27]. They show that the early lineages prior to and around the time of the Great Oxygenation Event were likely unicellular, freshwater, had small cell diameters (less than 2.5 *μm*) and lacked the ability to fix nitrogen. The analyses here did not find evidence for an early origin of multicellularity (i.e. filamentous morphologies; [84, 85]). The latter interpretation we show was likely a result of a long branch attraction artifact that placed *Pseudanabaena* clade, a group of filamentous taxa, branching early in their trees. The current study examines a much larger number of taxa and a much larger of characters. It confirms that ASR can lead to new insights in the evolution of traits that are not fossilizable, such as the likely presence of particular ecological and metabolic traits at various intervals in geologic history.

Before the GOE, ASR infers that Cyanobacteria were terrestrial (freshwater, low salinity) had small cell diameters (<2.5*μm*), were unicellular, non-motile, grew by binary fission in a single plane, did not fix nitrogen, were benthic or sessile (not planktonic), and may have had an extracellular sheath and lived in an epilithic or endolithic association with rocky substrates. Thus, these organisms were likely living at the bottom of shallow lakes or streams, and may have had transient emergence onto dry land.

Immediately after the GOE, a number of fundamentally new sets of traits appeared. Such new traits include filamentous growth (with isopolar filaments), confirmed extracellular sheath and epi/endolithic growth, and cell diameters beginning to increase to 3.5 *μm*. Additional innovations include the appearance of hormogonia (aiding dispersal) and uniseriate trichomes. We interpret this to be an expanded colonization of terrestrial (low salinity) environments, perhaps even onto dry land surfaces, possibly creating thin algal crusts. Interestingly, this also appears to be a time when a number of fundamental, new lineages branch off the tree. If this is an adaptive radiation in terrestrial ecosystems, it could explain why branching relationships in this part of the tree are difficult to resolve.

In the earliest Mesoproterozoic, a number of new lineages and traits likely appeared. These traits include: fission in multiple planes, akinetes, heterocysts, nitrogen fixation, gliding motility (again aiding dispersal across surfaces) appearing in multiple clades nearly simultaneously, and the first appearance of planktonic species. Most of these traits are inferred to have occurred in freshwater ancestors, however ASR suggests that this might be a time when marine clades also begin to appear. Most of the marine clades appear to be associated with surfaces (having filamentous morphologies and gliding motility). Around 1.4 Ga, false branching is first seen, along with heteropolar filaments (again in freshwater environments). The first confirmed marine clade seen in the tree is 1.1 Ga, and was likely benthic or sessile. Towards the end of the Mesoproterozoic, ASR predicts the appearance of multitrichomous filaments. Thus, while the Mesoproterozoic is often referred to as the “boring billion” or the “barren billion” ([94, 95]), we show that there are likely significant evolutionary changes occurring in oxygenic phototrophs, but that these changes have largely gone unnoticed or under-reported because most sedimentary deposits are marine in origin.

The Neoproterozoic appears to be a time when a number of planktonic freshwater and marine clades originate. After the appearance of planktonic growth, one then sees planktonic nitrogen-fixing followed by planktonic nitrogen-fixing marine species occurring in the Phanerozoic. We also see late origins of baeocytes, as well as a number of other traits in the tips of the trees.

A key evolutionary pattern, which has now been seen in a growing number of studies, is that much of the evolutionary history of Cyanobacteria has most likely existed in freshwater ecosystems. Nearly all of the gains of traits considered to be unique in the Cyanobacteria (sheathed filaments, hormogonia, true and false branching, akinetes, and heterocysts, nitrogen fixation) all likely first appeared in terrestrial ancestral nodes, including the endosymbiosis of early chloroplasts ([27]). Both Cyanobacteria and eukaryotic algae appear to have only been in freshwater ecosystems for much of Earth’s history. Thus, any biogeochemical processes (such as oxidative weathering prior to the GOE) must consider that the major activities of oxygenic phototrophs at the time were likely terrestrial (and not marine) processes.

## Conclusions

Our combined analysis of character state evolution across the cyanobacterial phylogeny show evidence for the acquisition of a suite of morphological ecological characters at the end of the Paleoproterozoic and into the Mesoproterozoic. Prior to the Mesoproterozoic, rates of character transitions were elevated and likely represents an adaptative radiation of cyanobacterial species across niche space in freshwater habitats. We reconstruct early species as unicellular with small cell diameter and benthic/planktonic habit, later gaining freshwater filamentous species that have hormogonia, begin to fix nitrogen, and have larger cell diameters. Through the Mesoproterozoic, we observe relative stasis and a decrease in evolutionary rates for most traits. Only in the Neoproterozoic and Phanerozoic are marine and planktonic species seen. In association with the gain of these characters, we observe some support for an additional rate shift localized between 0.8 and 0.5 Ga, though simulation studies suggest this may be an artifact of a coincident diversification event. Phylogenomic-based character studies such as ours are revealing a nuanced view of character evolution that cannot be gleaned from analysis of the geologic fossil record alone. We demonstrate that a well-resolved phylogeny and phenotypic data from extant taxa can provide key insights into the evolutionary history of Cyanobacteria and the history of our planet.

## Supporting Information

S1 Methods (PDF)

S1 Results (PDF)

S1 Fig Sequence alignment of tRN-Leu_*UAA*_. (PDF)

S2 Fig Sequence alignment of DnaE. (PDF)

S3 Fig Ancestral State Reconstruction of Growth Habit Showing Shift in Ages Given Different Root Maximum Age Assumptions. (PDF)

S4 Fig Likelihood profile plots for each trait fit independently with the epoch model. Plots are ordered by the location of their peak. Scale is indicated by horizontal black dotted lines. Arrows placed at peak indicate whether the rates for 0 → 1 and 1 → 0 transitions decrease (blue, down) or increase (red, up) after the shift, at the most likely shift location. Thickness of the line is proportional to the log of the shift magnitude. Red dashed lines indicate the location of the two peaks in the cumulative sum across traits (1.7 and 0.6 Ga).
(PDF)

S5 Fig Likelihood profile plots for simulation study vs. the empirical data. Green lines indicate the simulated data where 25 trait datasets were randomly aggregated and averaged. Dark green line indicates average profile across all simulations. Red lines indicate empirical data, with light red lines indicating individual profiles for each of 20 trees from the posterior, and the dark green line indicating the average across trees.
(PDF)

S6 Fig Comparison of rate shift magnitudes from simulation study vs. empirical data. Warmer colors indicate higher density. For simulated and empirical data, we estimated for each trait the rate shift parameters (*r*_01_ and *r*_10_) across a sequence of shift times from the root of the tree (2.7 Ga) to the present. We then used kernel density estimation to determine whether downshifts or upshifts in trait evolution were more common. The magnitude of the rate shift is expressed in log units, with a value of 0 indicating no rate shift (i.e. the transition rate parameters *q*_01_ and *q*_10_ are equal before and after the shift). Note that the simulated data is biased prior to around 1 Ga to give the appearance of accelerations of transition rates. However, we observe the opposite pattern in the empirical data, with most traits exhibiting a downshift prior to 1 Ga, and and accelerations afterward.
(PDF)

S7 Fig Ancestral State Reconstruction of Heterocysts and Akinetes. (PDF)

S8 Fig Ancestral state reconstruction of fission in multiple planes and multi-trichomous filaments. (PDF)

S9 Fig Ancestral state reconstruction of uniseriate and multiseriate trichomes. (PDF)

S10 Fig Ancestral state reconstruction of true branching and false branching. (PDF)

S11 Fig Ancestral state reconstruction of hormogonia and gas vesicles. (PDF)

S12 Fig Ancestral state reconstruction of isopolar and heteropolar filaments. (PDF)

S13 Fig Ancestral state reconstruction of cell diameter with different thresholds. (PDF)

S14 Fig Ancestral state reconstruction of epi/endolithic and the extracellular sheath. (PDF)

S15 Fig Ancestral state reconstruction of nitrogen fixation and microbial mats. (PDF)

S16 Fig Ancestral state reconstruction of motility and gliding motility. (PDF)

S1 Table Epoch model fits and phylogenetic signal for phylogenies time-calibrated to a maximum root age of 3.8 Ga. (PDF)

S2 Table Ranges for estimated node ages^†^ for major cyanobacterial clades from different taxon sets. (PDF)

S1 File Genbank accession numbers for data used in this study. (CSV)
