# Supplementary material for: A Comprehensive Study of Cyanobacterial Morphological and Ecological Evolutionary Dynamics through Deep Geologic Time
Source: PLoS One. 2016 Sep 20;11(9):e0162539. doi: 10.1371/journal.pone.0162539 (PMC5029880; doi:10.1371/journal.pone.0162539)
Supplement: S1 Methods — (PDF) [file pone.0162539.s001.pdf]

Supplementary Information: S1 Methods

We conducted a simulation study to determine whether common types of phylogenetic error likely to be present in our dataset were likely to artifactually result in support for a rate shift at the time intervals we observed in our dataset. We tested whether variation among our estimated phylogenies and taxon sets (including artifacts introduced by long-branch attraction) resulted in predictable or systematic types of error in our estimation of shifts in trait evolution. To test this, we simulated constant-rate Mk2 model on one phylogeny and re-estimated parameters under both the constant-rate and epoch models on another phylogeny. To conduct the study, we collected trees from three taxon sets with known long-branch attraction artifacts, four taxon sets time-scaled with a maximum age constraint of the root at 3.8 billion years, and two taxon sets with the maximum age constraint at the root of 2.7 billion years. We obtained 100 trees from the posterior of each of these taxon sets, to produce a set of 900 potential trees for our simulation study. We then randomly selected trees from within this set and simulated the constant rate Mk2 model. We drew parameters for the each simulation by drawing from the set of estimated parameters from the true dataset across 40 different trees (without long-branch attraction artifacts) for each of the 25 different phenotypic traits (1000 unique parameter sets). We then randomly selected another phylogeny from this set to re-estimate under the constant rate and epoch models. We estimated the likelihood for 38 time slices along the sequence from 0 to the total length of the tree, (just was as done in the actual dataset) for the epoch model. We then summarized the results to see if the re-estimated profiles resembled our observed profile plots. We further explored the factors that result in higher likelihood support for the epoch model (Type I error) by using multiple regression on our simulation results. For each simulated dataset, we obtained 4 different dependent variables: the maximum support for the epoch model over the constant rate model (maxL), the timing of the shift at the maximum support value (maxT), the value of the likelihood support for a shift at 1.6 Ga (L1.7), and the likelihood support for a shift at 0.6 Ga (L0.6). Each dependent variable was tested using forward and backwards stepwise AIC with the following full model:

$$E[y] = q_{01} + q_{10} + q_{01} * q_{10} + TL + T_{dist} + T_{cat} + T_{match} + T_{dist} * q_{01} * q_{10} \quad (1)$$

Where q_{01} and q_{01} are the true values of the log-transformed transition rates from $0 \to 1$ and $1 \to 0$ respectively, TL is the difference in tree length between the true tree and the estimating tree, T_{dist} is the geodesic distance between trees calculated using the *dist.multiPhylo* function of the R package *distory* [1], T_{cat} is a categorical variable indicating which combination of the 9 posteriors were used for the simulating and estimating trees, and T_{match} is an indicator variable indicating if the simulating and estimating trees are obtained from the same posterior.

References

1. Chakerian J, Holmes S. distory: Distance Between Phylogenetic Histories; 2013. R package version 1.4.2. Available from: `http://CRAN.R-project.org/package=distory`.
