# Supplementary material for: A Comprehensive Study of Cyanobacterial Morphological and Ecological Evolutionary Dynamics through Deep Geologic Time
Source: PLoS One. 2016 Sep 20;11(9):e0162539. doi: 10.1371/journal.pone.0162539 (PMC5029880; doi:10.1371/journal.pone.0162539)
Supplement: S1 Results — (PDF) [file pone.0162539.s002.pdf]

Supplementary Information: S1 Results

Summary of Taxon Addition and Subtraction Analyses. Analyses were carried out using Maximum Parsimony first and were followed up using a smaller number of analyses using MrBayes (which were computationally intensive, given the size of the dataset, where a single analysis could take a week running on multiple nodes on the XSEDE server). Much of the LBA conflicts were between long branches that diverged in a similar part of the tree, which are fundamentally difficult to resolve. A summary of the analyses follows focusing on each rogue taxon.

Octopus Spring clade: This was a robust clade, branching between *Gloeobacter* and SynPro in the core cyanobacterial tree (Fig. 2). Most phylogenies showed this clade in this location, however inclusion of this clade can induce LBA artifacts with other rogue taxa. For example, when this clade and the *Pseudanabaena* clade were analyzed, it was pulled higher up in the tree to branch near the *Pseudanabaena*. This clade exhibited LBA in the presence of the *Pseudanabaena* clade and sometimes in the presence of *Oscillatoriales* JSC-12. Branching behavior was unaffected by the presence of *Geitlerinema* PCC 7407, *Geitlerinema* 7105, and *Prochlorothrix hollandica*. The taxa in this clade lack the tRNA-Leu$_{UAA}$ intron and lack the DnaE intein (S2 Fig). This is consistent with it branching deep in the Cyanobacteria, since the early lineages lack these rare genetic features.

Prochlorothrix hollandica: This lineage branched between the SynPro clade and the LPP-B clade in the (Fig. 2). Most phylogenies with individual rogue taxa showed this lineage branching in this location, however inclusion of the *Pseudanabaenas* could lead to *Prochlorothrix* branching sister to SynPro, with the *Pseudanabaena* clade branching deep or higher up in the tree in an anomalous position (see below). This lineage sometimes exhibited long branch attraction (LBA) in the presence of the *Pseudanabaena* clade. Branching behavior was unaffected by the presence of the *Geitlerinema* 7407, *Geitlerinema* 7105, the *Synechococcus elongatus* clade, the Octopus Spring clade, core tree (Fig. 2) and *Oscillatoriales* cyanobacterium JSC-12. This taxon has the tRNA-Leu intron (S1 Fig) and lacks the DnaE inteins (S2 Fig). The presence of the intron is inconsistent with branching sister to SynPro, and is more consistent with branching between SynPro and the LPP-B clade (the latter of which also have the intron).

Pseudanabaena clade: This was a robust clade (with respect to branching relationships within the clade), branching between SynPro and LPP-B in the core tree (Fig. 2). Many analyses with addition of other rogue taxa showed this clade branching in various anomalous positions. In some analyses, the *Pseudanabaenas* branched deep in the core tree (also resulting in an anomalous core cyanobacterial phylogeny). This clade exhibited LBA in the presence of *Prochlorothrix hollandica*, the Octopus Spring clade, and the *Synechococcus elongatus* clade. Branching behavior was unaffected by the presence of *Geitlerinema* PCC 7105, *Geitlerinema* PCC 7407 and the *Oscillatoriales* cyanobacterium JSC-12. *Pseudanabaena* PCC 403 and *Pseudanabaena* PCC 7367 have the tRNA-Leu intron (S1 Fig) and *Synechococcus* PCC 7502 and *Pseudanabaena biceps* PCC 7429 have both the DnaE intein-1 and the split DnaE intein (S2 Fig). The presence of these rare genetic features (which are not found in the early cyanobacterial lineages) supports branching of this group higher up in the tree, and is inconsistent with this clade branching deep in the Cyanobacteria basal to the SynPro divergence.

Synechococcus elongatus clade: This was a robust clade, branching between SynPro and LPP-B in the core cyanobacterial tree (Fig. 2). In most analyses with other rogue taxa, this clade branched in the same location as in the core cyanobacterial tree. However, in the presence of the *Pseudanabaenas* this clade branched sister to SynPro. Branching behavior was unaffected by the presence of *Prochlorothrix hollandica*, *Oscillatoriales* JSC-12, *Geitlerinema* 7105 and *Geitlerinema* PCC 7407. The taxa in this clade have the tRNA-Leu intron (S1 Fig) and have the DnaE split intein. The

presence of both of these rare genetic traits in inconsistent with branching sister to SynPro, and is consistent with its branching between SynPro and LPP-B (the latter of which also has both rare genetic events). A study of operon conservation patterns showed that *Synechococcus elongatus* has an operon characteristics more like taxa in the SPM and NX clades [1], rather than the SynPro clade, again supporting branching position between SynPro and LPP-B.

Geitlerinema PCC 7407: This lineage branched between LPP-B and the AcTh clades in the core cyanobacterial tree. This lineage exhibited LBA in the presence of the *Pseudanabaena* clade and *Geitlerinema* PCC 71105. Branching behavior was unaffected by the presence of the Octopus Spring clade, the *Synechococcus elongatus* clade, *Prochlorothrix hollandica*, and *Oscillatoriales* cyanobacterium JSC-12. This taxon has the tRNA-Leu intron (S1 Fig) and has the split DnaE intein (S2 Fig).

Oscillatoriales cyanobacterium JSC-12: This lineage branched between AcTh and the *Oscillatoriales sensu stricto* in the core cyanobacterial tree. This lineage was found to be quite sensitive to the presence of other rogue taxa in the analysis, branching in various anomalous positions (between LPP-B and AcTh or sister to AcTh) and inducing anomalous branching positions in other rogue taxa. This lineage exhibited LBA in the presence of *Geitlerinema* PCC 7407 and *Geitlerinema* 7105 and various combinations of rogue taxa. Branching behavior was unaffected by the presence of the *Pseudanabaena* clade, the *Synechococcus elongatus* clade, the Octopus Spring clade, and *Prochlorothrix hollandica*. This taxon has the tRNA-Leu intron (S1 Fig) and lacks the DnaE intein (S2 Fig).

Geitlerinema PCC 7105: This lineage branched between AcTh and the *Oscillatoriales sensu stricto* in the core cyanobacterial tree. This lineage exhibited LBA in the presence of *Oscillatoriales* JSC-12 (branching between LPP-B and AcTh). Branching behavior was unaffected by the presence of *Geitlerinema* PCC 7407, the *Pseudanabaena* clade, the Octopus Spring clade, the *Synechococcus elongatus* clade, and *Prochlorothrix hollandica*. This taxon lacks the tRNA-Leu intron and lacks the DnaE intein (S2 Fig).

Simulation study of epoch models. For the dependent variables *maxT, maxL, L1.6* and *L0.6*, the only common explanatory factors in the best-fitting model were the transition rates and their interaction (q_{01}, q_{10} and $q_{01} * q_{10}$). For *maxL* and *L0.6*, no other variable was included in the model. For *maxT* and *maxL*, the variable T_{match} was included in the final model, but not significant at the 0.05-level (p = 0.06 and 0.15, respectively). *L1.6* also included T_{dist}, but again, was not significant (p = 0.13).

Tree distance played virtually no role in affecting any of the four explanatory variables. This indicates that the likelihood profiles observed in the simulation study could be obtained even when the phylogenies are quite similar. Rather, only the model generating parameters affect the strength of support for the epoch model, and the location of the shift. The main effects of higher transition rates, in general, was to lower values of *maxL*, *L*1.6 and *L*0.6. However the interaction reversed the effect, and when both of these values were large, the three variables increased. Similarly, the main effects for transition rates predicted maximum likelihood shift locations (*maxT*) that were more recent with increasing rates, but the interaction suggested older shifts would be identified when both transition rates were large.

References

1. Memon D, Singh AK, Pakrasi HB, Wangikar PP. A global analysis of adaptive evolution of operons in Cyanobacteria. Antonie Van Leeuwenhoek. 2013;103(2):331–346.

Supporting Information

S1 Methods

S1 Results

S1 Fig

S2 Fig

S3 Fig

S4 Fig

S5 Fig

S6 Fig

S7 Fig

S8 Fig

S9 Fig

S10 Fig

S11 Fig

S12 Fig

S13 Fig

S14 Fig

S15 Fig

S16 Fig

S1 Table

S2 Table
