# Supplementary figures and images for: A Comprehensive Study of Cyanobacterial Morphological and Ecological Evolutionary Dynamics through Deep Geologic Time
Source: PLoS One. 2016 Sep 20;11(9):e0162539. doi: 10.1371/journal.pone.0162539 (PMC5029880; doi:10.1371/journal.pone.0162539)

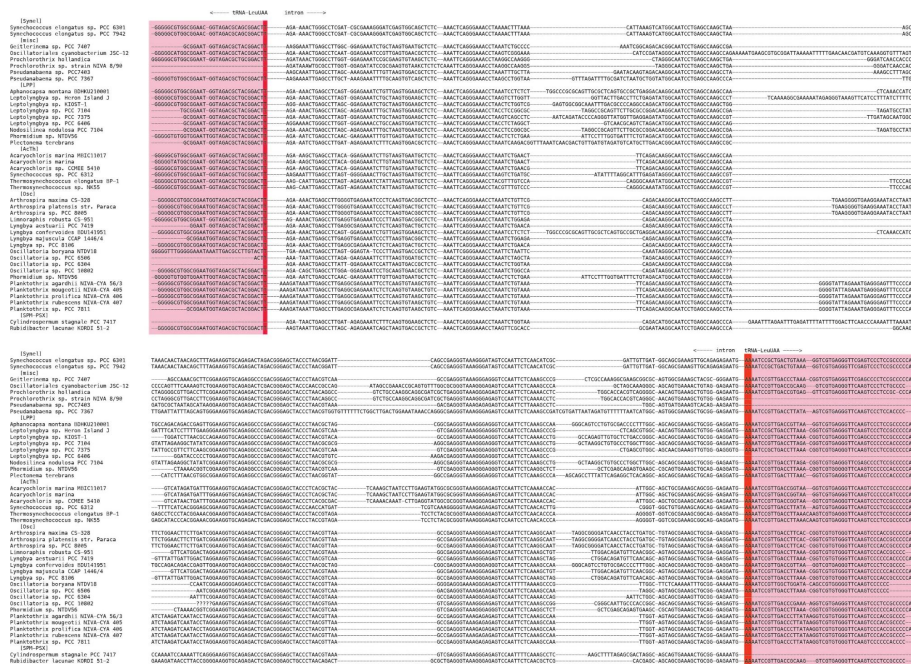

**S1 Fig. Sequence alignment of tRN-Leu<sub>UAA</sub>.**

Supplement: S1 Fig — (PDF) [file pone.0162539.s003.pdf]

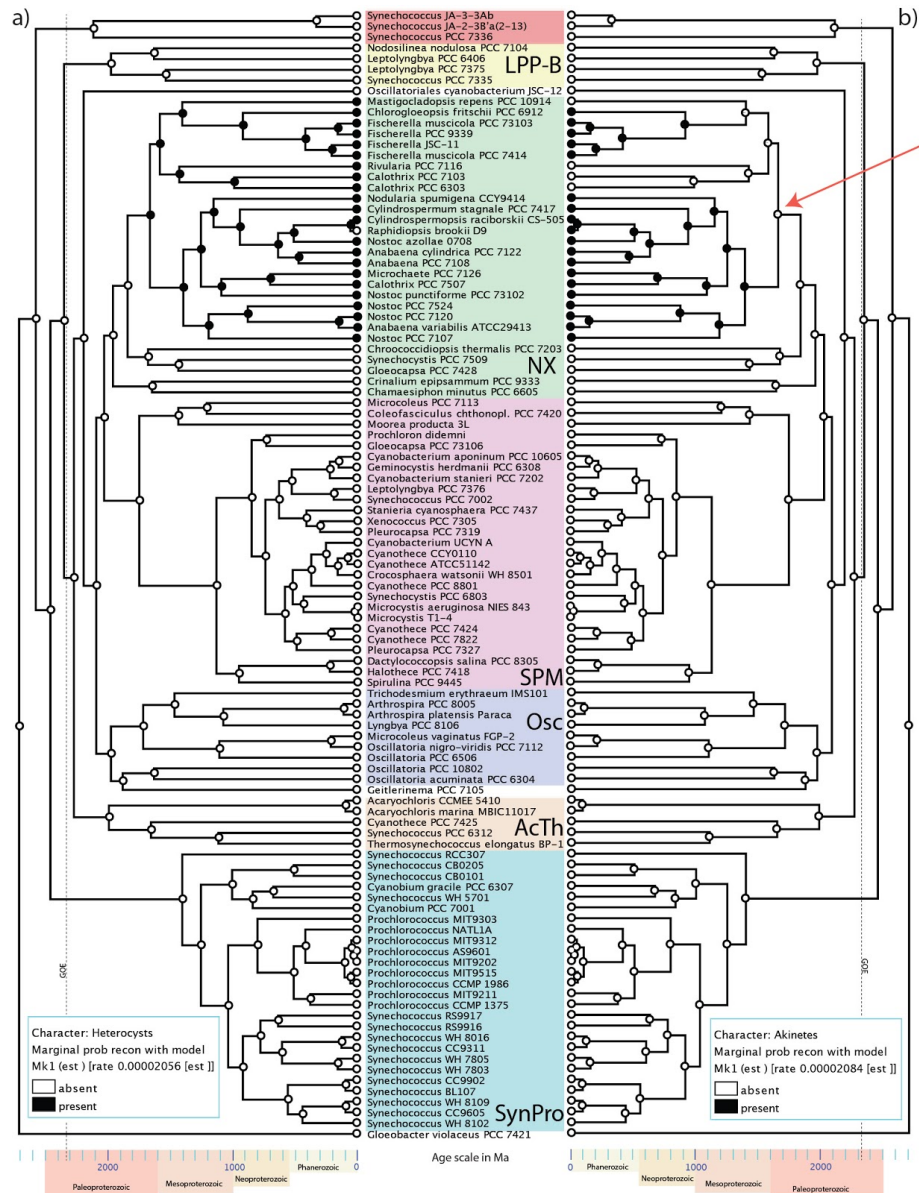

S7 Fig. Ancestral state reconstruction of heterocysts and akinetes.

Supplement: S7 Fig — (PDF) [file pone.0162539.s009.pdf]

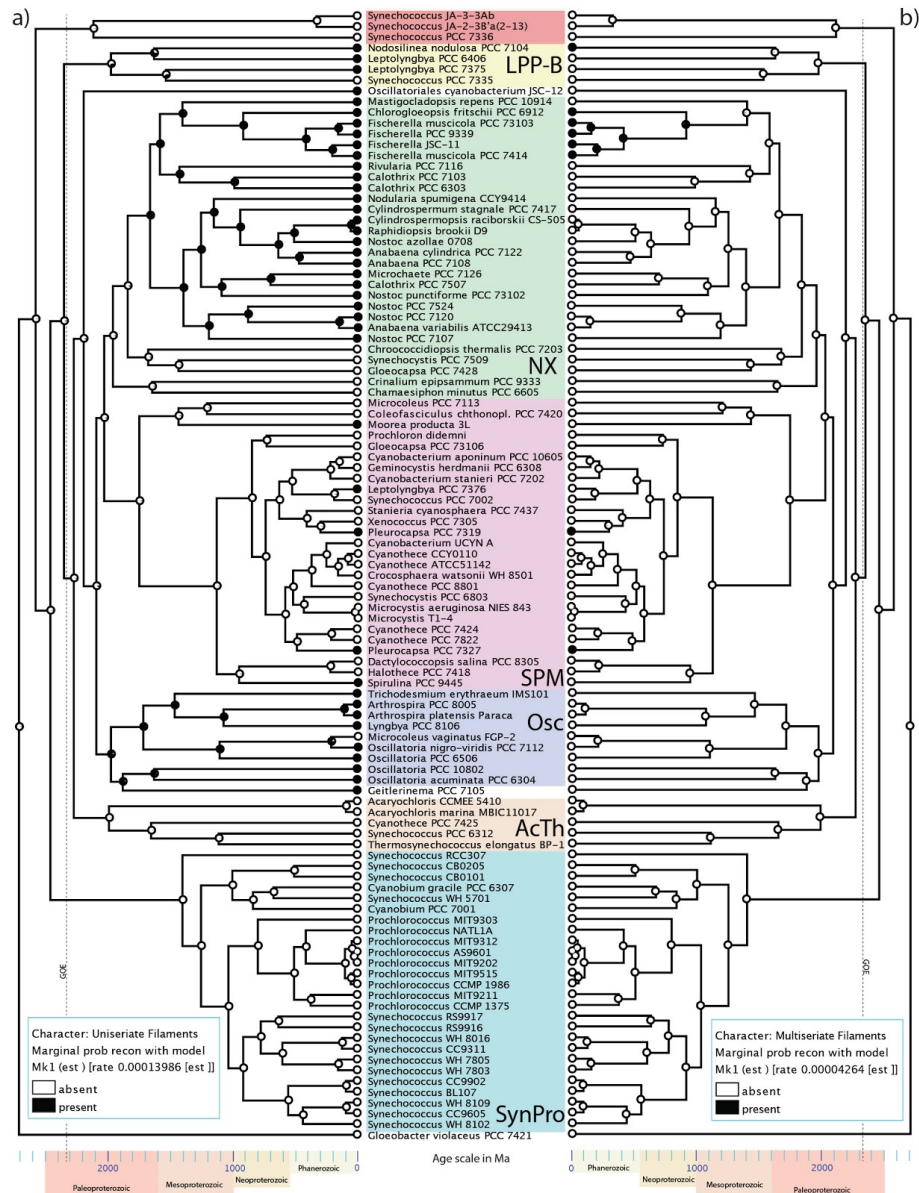

S9 Fig. Ancestral state reconstruction of uniseriate and multiseriate trichomes.

Supplement: S9 Fig — (PDF) [file pone.0162539.s011.pdf]

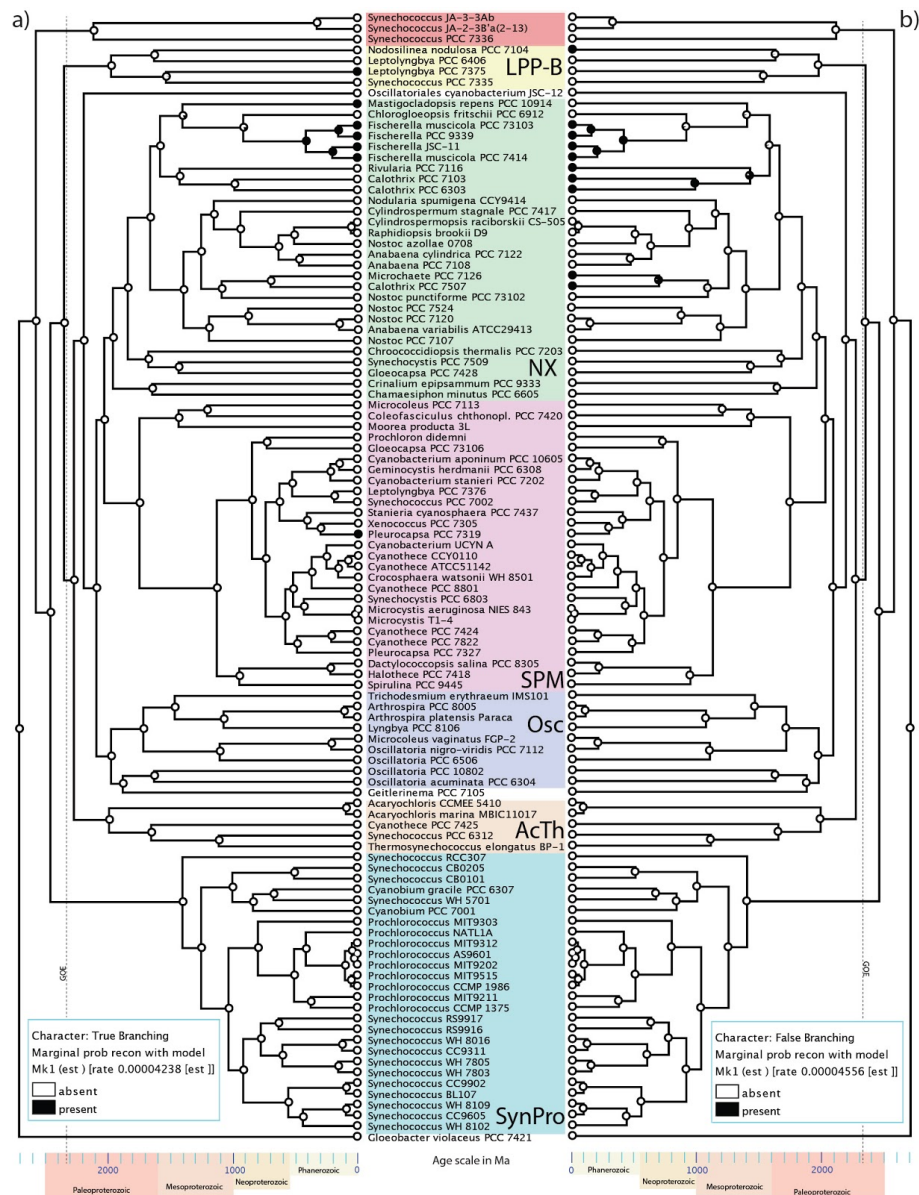

**S10 Fig. Ancestral state reconstruction of true branching and false branching.**

Supplement: S10 Fig — (PDF) [file pone.0162539.s012.pdf]

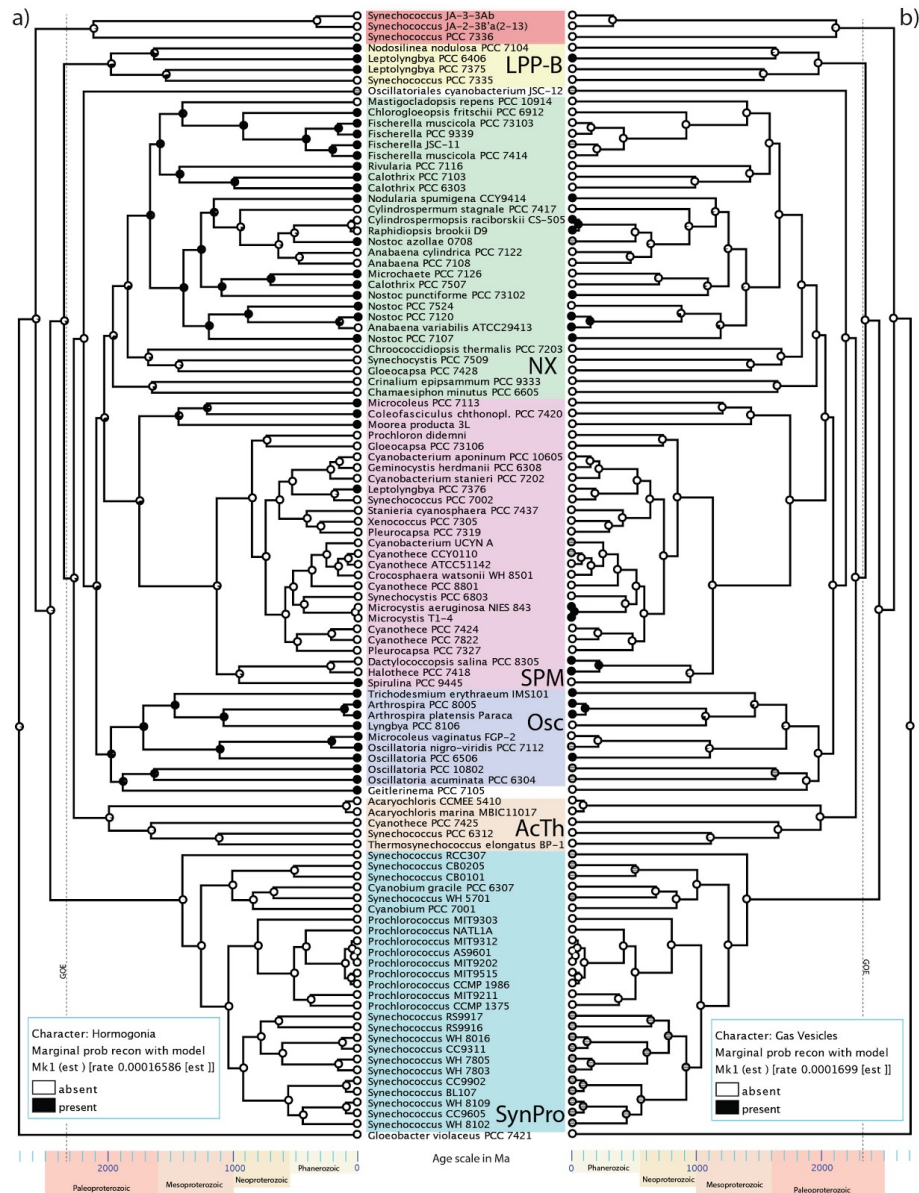

S11 Fig. Ancestral state reconstruction of hormogonia and gas vesicles.

Supplement: S11 Fig — (PDF) [file pone.0162539.s013.pdf]

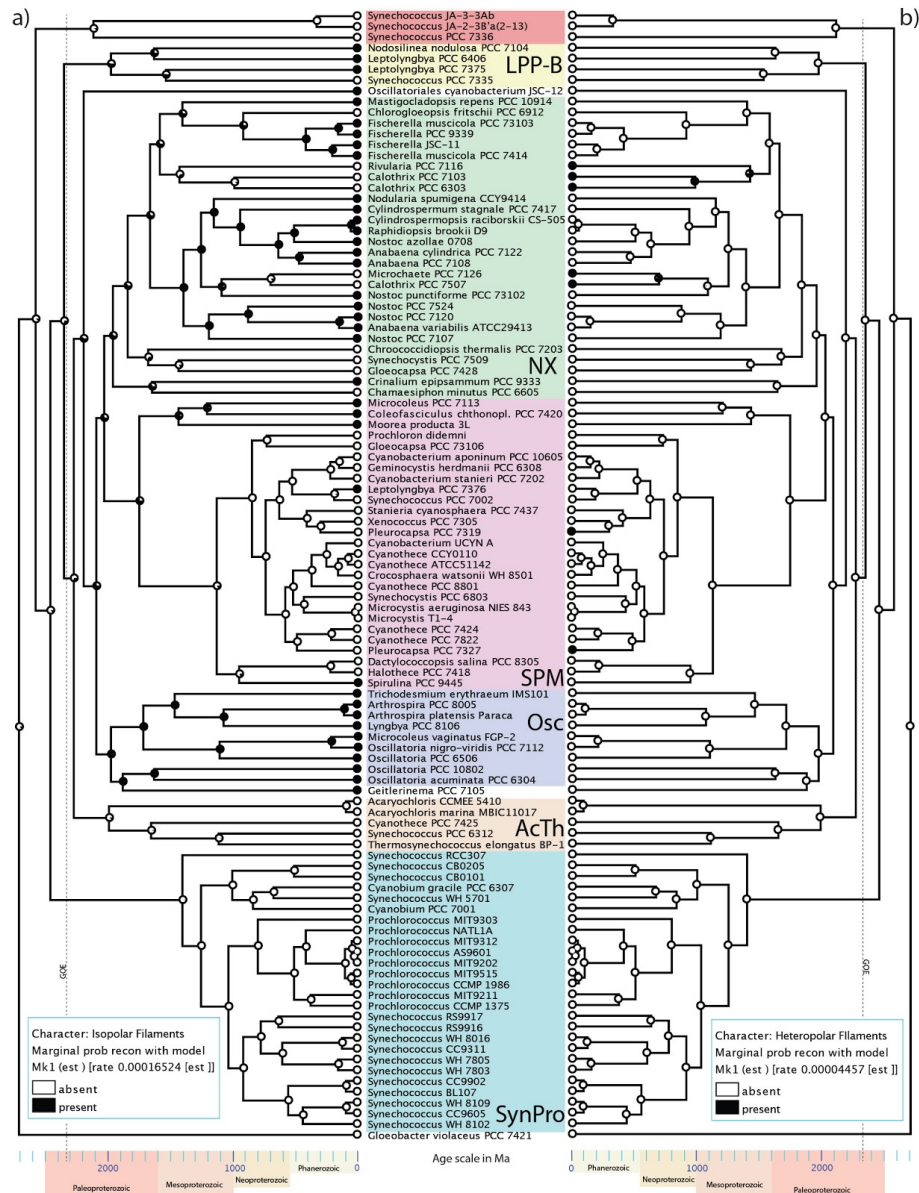

S12 Fig. Ancestral state reconstruction of isopolar and heteropolar filaments.

Supplement: S12 Fig — (PDF) [file pone.0162539.s014.pdf]

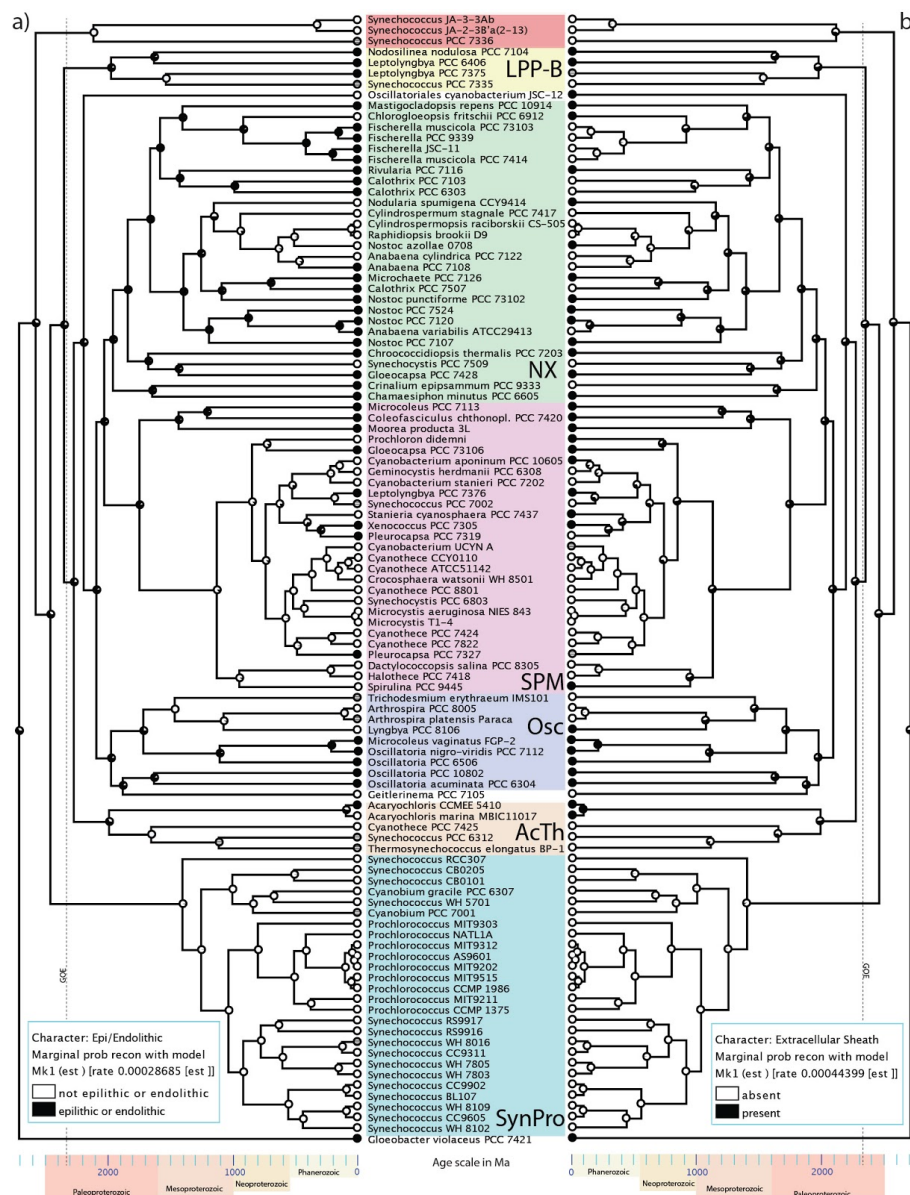

**S14 Fig.** Ancestral state reconstruction of epi/endolithic and the extracellular sheath.

Supplement: S13 Fig — (PDF) [file pone.0162539.s015.pdf]

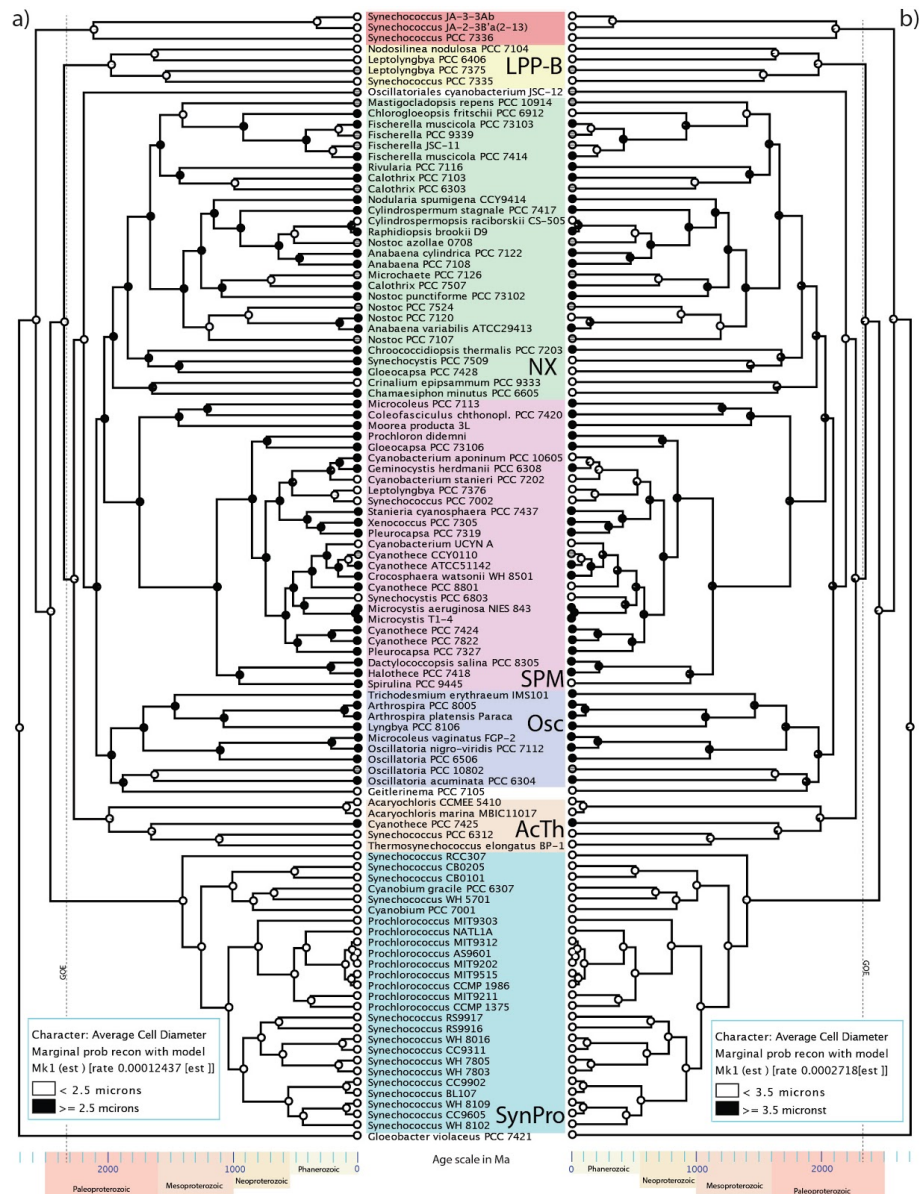

S13 Fig. Ancestral state reconstruction of cell diameter with different thresholds.

Supplement: S14 Fig — (PDF) [file pone.0162539.s016.pdf]

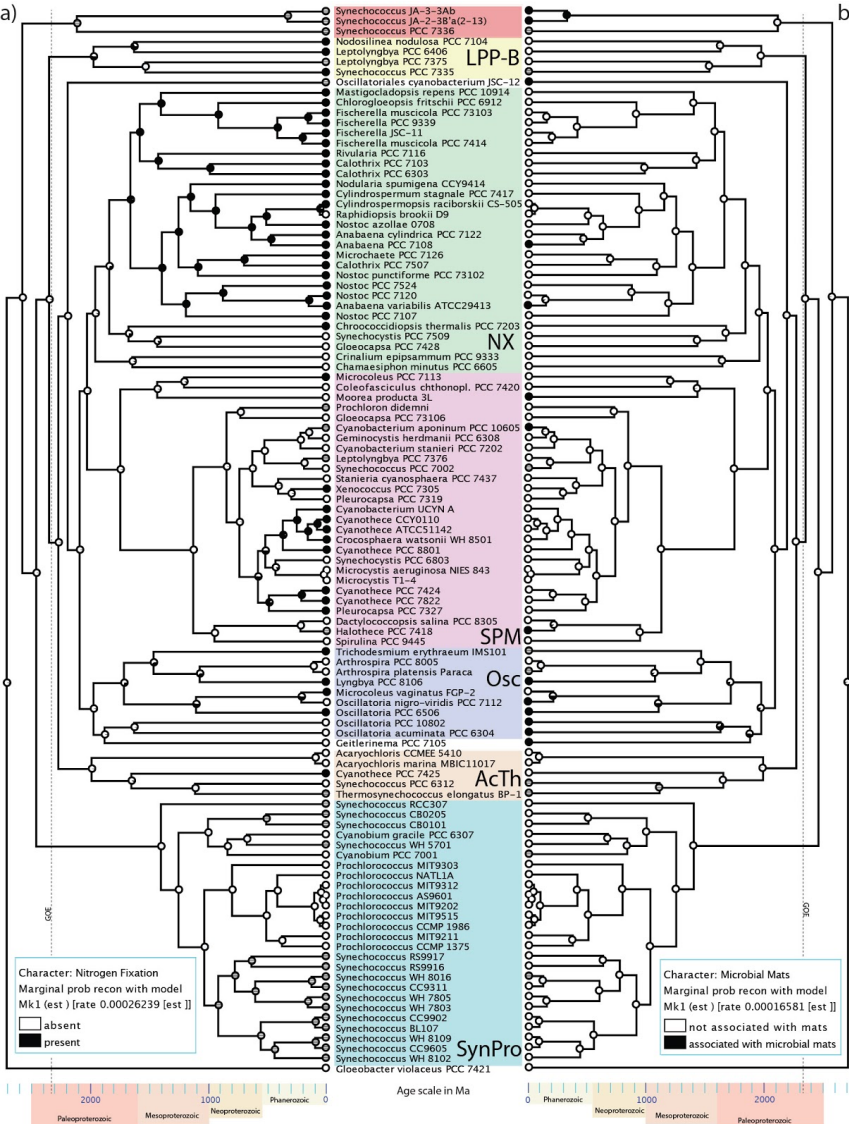

**S15 Fig. Ancestral state reconstruction of nitrogen fixation and microbial mats.**

Supplement: S15 Fig — (PDF) [file pone.0162539.s017.pdf]

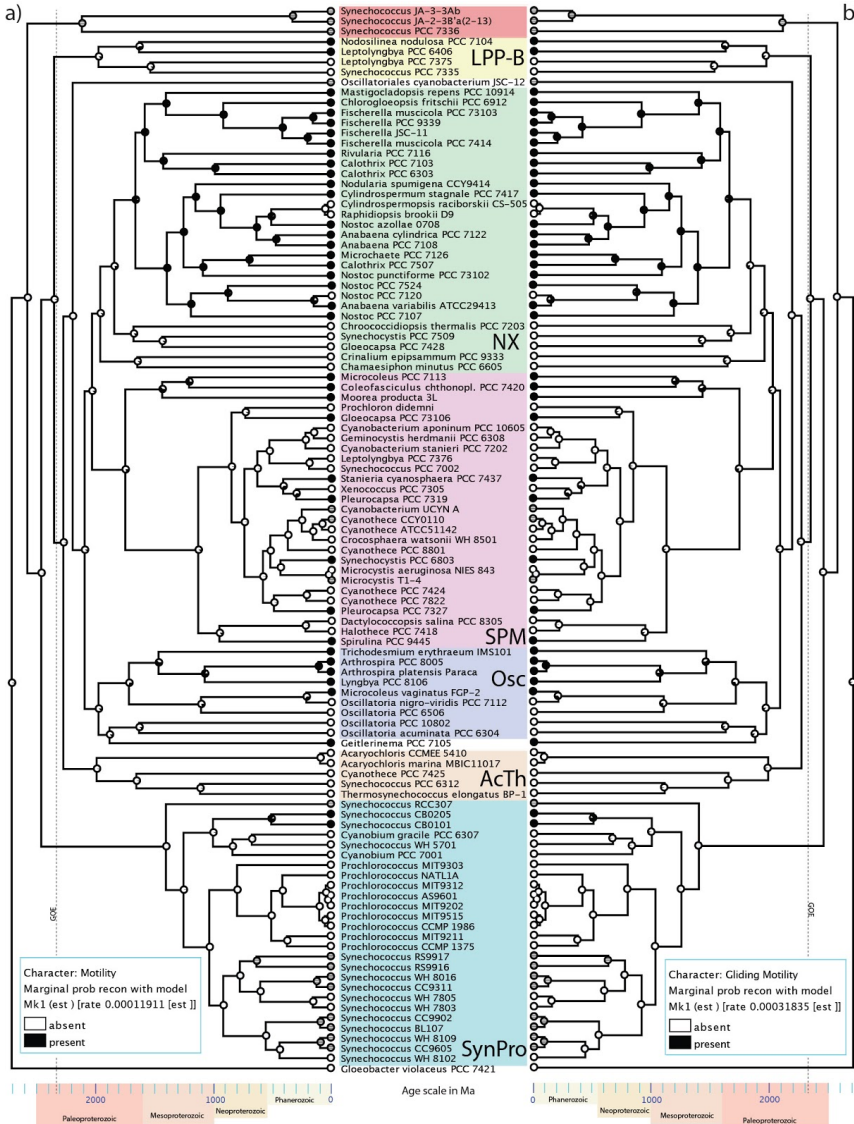

**S16 Fig. Ancestral state reconstruction of motility and gliding motility.**

Supplement: S16 Fig — (PDF) [file pone.0162539.s018.pdf]
