# Supplementary material for: A Comprehensive Study of Cyanobacterial Morphological and Ecological Evolutionary Dynamics through Deep Geologic Time
Source: PLoS One. 2016 Sep 20;11(9):e0162539. doi: 10.1371/journal.pone.0162539 (PMC5029880; doi:10.1371/journal.pone.0162539)
Supplement: S4 Fig — Plots are ordered by the location of their peak. Scale is indicated by horizontal black dotted lines. Arrows placed at peak indicate whether the rates for 0 → 1 and 1 → 0 transitions decrease (blue, down) or increase (red, up) after the shift, at the most likely shift location. Thickness of the line is proportional to the log of the shift magnitude. Red dashed lines indicate the location of the two peaks in the cumulative sum across traits (1.7 and 0.6 Ga). (PDF) [file pone.0162539.s006.pdf]

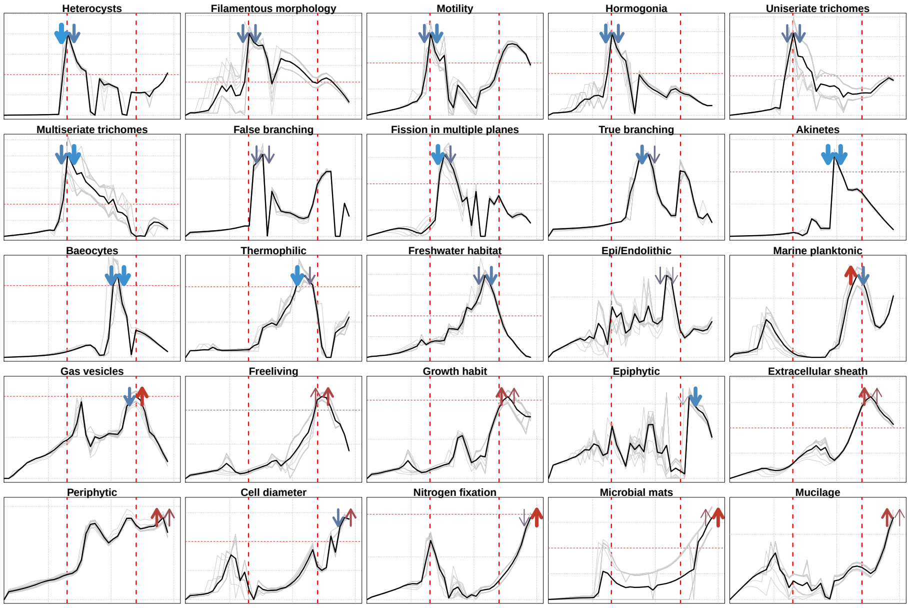

S4 Fig. Likelihood profile plots for each trait fit independently with the epoch model. Plots are ordered by the age of their maximum likelihood peak. Scale is indicated by horizontal black dotted lines. Arrows placed at peak indicate whether the rates for $0 \rightarrow 1$ and $1 \rightarrow 0$ transitions decrease (blue, down) or increase (red, up) after the shift, at the most likely shift location. Thickness of the line is proportional to the log of the shift magnitude. Red dashed lines indicate the location of the two peaks in the cumulative sum across traits (1.7 and 0.6 Ga).
