# Supplementary material for: A Comprehensive Study of Cyanobacterial Morphological and Ecological Evolutionary Dynamics through Deep Geologic Time
Source: PLoS One. 2016 Sep 20;11(9):e0162539. doi: 10.1371/journal.pone.0162539 (PMC5029880; doi:10.1371/journal.pone.0162539)
Supplement: S5 Fig — Green lines indicate the simulated data where 25 trait datasets were randomly aggregated and averaged. Dark green line indicates average profile across all simulations. Red lines indicate empirical data, with light red lines indicating individual profiles for each of 20 trees from the posterior, and the dark green line indicating the average across trees. (PDF) [file pone.0162539.s007.pdf]

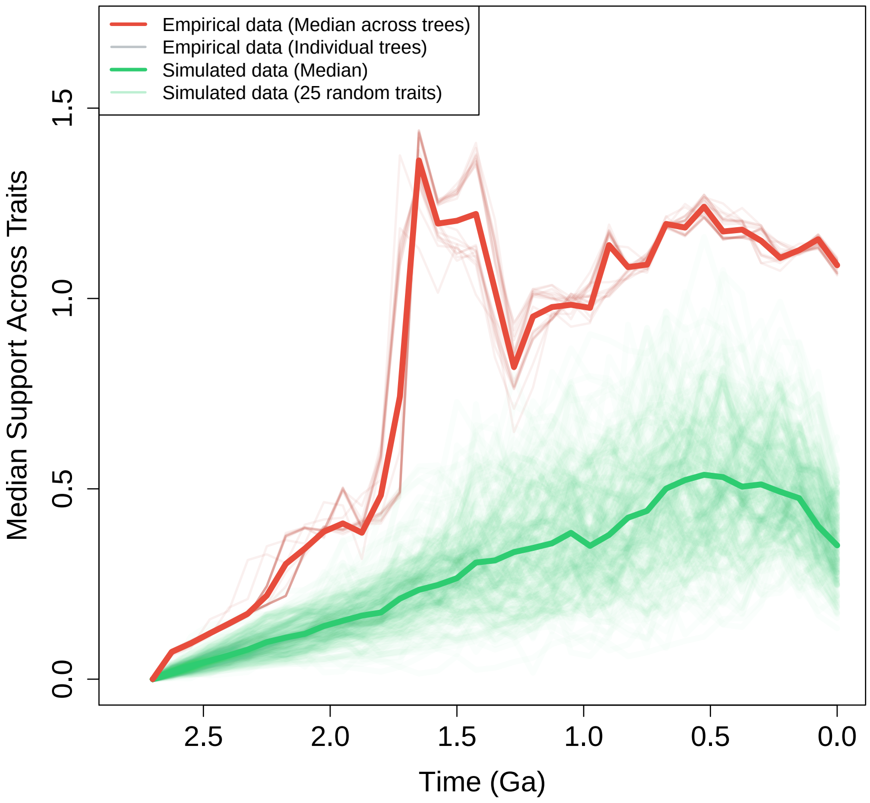

S5 Fig. Likelihood profile plots for simulation study vs. the empirical data. Green lines indicate the simulated data where 25 trait datasets were randomly aggregated and averaged. Dark green line indicates average profile across all simulations. Red lines indicate empirical data, with light red lines indicating individual profiles for each of 20 trees from the posterior, and the dark green line indicating the average across trees.
