# Supplementary material for: A Comprehensive Study of Cyanobacterial Morphological and Ecological Evolutionary Dynamics through Deep Geologic Time
Source: PLoS One. 2016 Sep 20;11(9):e0162539. doi: 10.1371/journal.pone.0162539 (PMC5029880; doi:10.1371/journal.pone.0162539)
Supplement: S6 Fig — Warmer colors indicate higher density. For simulated and empirical data, we estimated for each trait the rate shift parameters (r01 and r10) across a sequence of shift times from the root of the tree (2.7 Ga) to the present. We then used kernel density estimation to determine whether downshifts or upshifts in trait evolution were more common. The magnitude of the rate shift is expressed in log units, with a value of 0 indicating no rate shift (i.e. the transition rate parameters q01 and q10 are equal before and after the shift). Note that the simulated data is biased prior to around 1 Ga to give the appearance of accelerations of transition rates. However, we observe the opposite pattern in the empirical data, with most traits exhibiting a downshift prior to 1 Ga, and and accelerations afterward. (PDF) [file pone.0162539.s008.pdf]

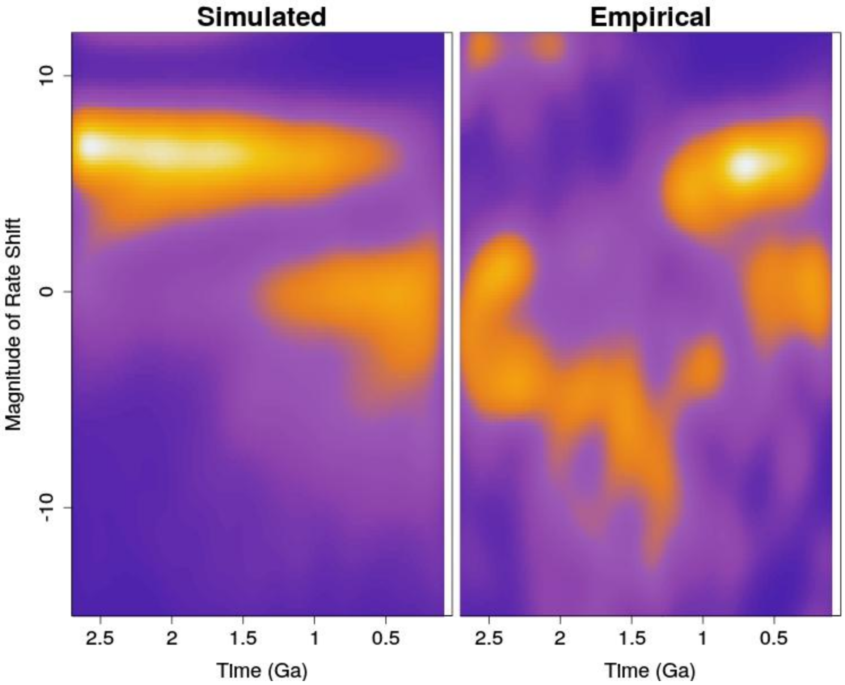

S6 Fig. Comparison of rate shift magnitudes from simulation study vs. empirical data. Warmer colors indicate higher density. For simulated and empirical data, we estimated for each trait the rate shift parameters (r_{01} and r_{10}) across a sequence of shift times from the root of the tree (2.7 Ga) to the present. We then used kernel density estimation to determine whether downshifts or upshifts in trait evolution were more common. The magnitude of the rate shift is expressed in log units, with a value of 0 indicating no rate shift (i.e. the transition rate parameters q_{01} and q_{10} are equal before and after the shift). Note that the simulated data is biased prior to around 1 Ga to give the appearance of accelerations of transition rates. However, we observe the opposite pattern in the empirical data, with most traits exhibiting a downshift prior to 1 Ga, and and accelerations afterward.
