# Supplementary material for: A Comprehensive Study of Cyanobacterial Morphological and Ecological Evolutionary Dynamics through Deep Geologic Time
Source: PLoS One. 2016 Sep 20;11(9):e0162539. doi: 10.1371/journal.pone.0162539 (PMC5029880; doi:10.1371/journal.pone.0162539)
Supplement: S8 Fig — (PDF) [file pone.0162539.s010.pdf]

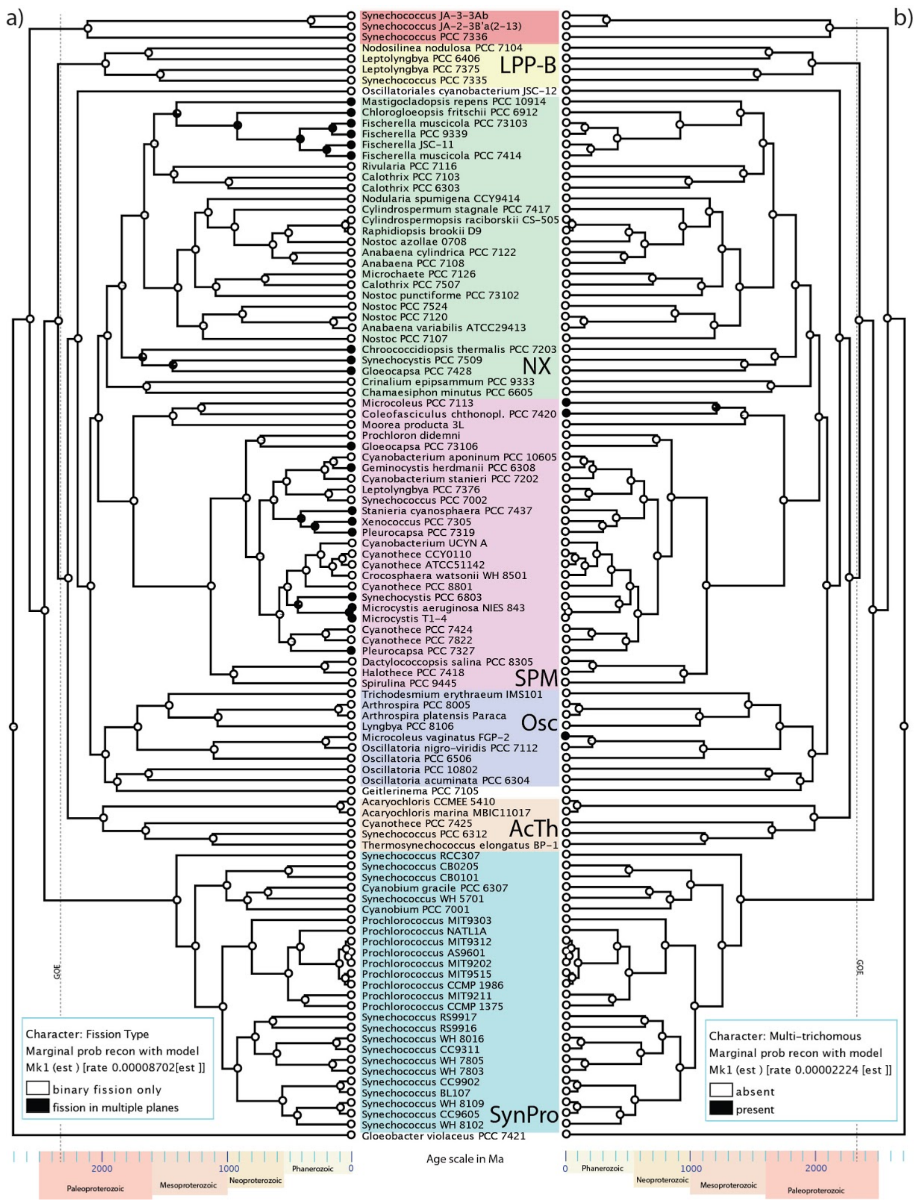

S8 Fig. Ancestral state reconstruction of fission in multiple planes and multi-trichomous filaments.
