# Supplementary material for: A Comprehensive Study of Cyanobacterial Morphological and Ecological Evolutionary Dynamics through Deep Geologic Time
Source: PLoS One. 2016 Sep 20;11(9):e0162539. doi: 10.1371/journal.pone.0162539 (PMC5029880; doi:10.1371/journal.pone.0162539)
Supplement: S1 Table — (PDF) [file pone.0162539.s019.pdf]

S1 Table. Epoch model fits and phylogenetic signal for phylogenies time-calibrated to a maximum root age of 3.8 Ga.

Trait	Δ lnL[‡]	Shift Age (Ga)[+]	Shift cluster
Filamentous morphology	4.06	1.8	Mesoproterozoic
Nitrogen fixation	1.69	1.8	Mesoproterozoic
Uniseriate trichomes	5.62	1.7	Mesoproterozoic
Hormogonia	3.05	1.7	Mesoproterozoic
Motility	3.00	1.7	Mesoproterozoic
Heterocysts	3.66	1.7	Mesoproterozoic
Fission in multiple planes	3.59	1.6	Mesoproterozoic
Gas vesicles	2.07	1.6	Mesoproterozoic
False branching	1.35	1.6	Mesoproterozoic
Freshwater habitat	1.53	1.1	
Baeocytes	3.02	1.0	
Akinetes	2.36	1.0	
Thermophilic	2.19	0.9	
Multiseriate trichomes	3.13	0.7	Neoproterozoic
Marine planktonic	1.65	0.6	Neoproterozoic
True branching	1.43	0.6	Neoproterozoic
Extracellular sheath	3.98	0.5	Neoproterozoic
Growth habit	1.65	0.6	Neoproterozoic
Free-living	2.64	0.5	Neoproterozoic
Epiphytic	0.57	0.5	Neoproterozoic
Periphytic	1.04	0.2	
Cell diameter	2.81	0.1	
Microbial mats	2.70	0.1	
Mucilage	1.53	0.1	

‡ Difference between best-fit epoch model and a constant rate model

[+] Age indicates the best estimate of the timing of a shift under an epoch model.
