# Supplementary material for: A Comprehensive Study of Cyanobacterial Morphological and Ecological Evolutionary Dynamics through Deep Geologic Time
Source: PLoS One. 2016 Sep 20;11(9):e0162539. doi: 10.1371/journal.pone.0162539 (PMC5029880; doi:10.1371/journal.pone.0162539)
Supplement: S2 Table — (PDF) [file pone.0162539.s020.pdf]

S2 Table. Ranges for estimated node ages† for major cyanobacterial clades from different taxon sets.

Clade	Age Constraints Minage	Maxage	Taxon Set 1	Taxon Set 2	Taxon Set 3	Taxon Set 4	Eon
Cyanobacteria	2.320	2.700	2.699‡	2.699‡	2.699‡	2.699‡	Archean
SynPro basal node			2.443±0.021	2.415±0.027	2.398±0.026	2.456±0.022	Paleoprot.
SynPro clade			1.410±0.171	1.378±0.162	1.256±0.152	1.430±0.146	Mesoprot.
Synechococcus-Prochlorococcus			0.982±0.171	0.953±0.164	0.889±0.144	0.1003±0.146	Neoprot.
Synechococcus			0.849±0.156	0.835±0.151	0.787±0.133	0.885±0.136	Neoprot.
Prochlorococcus			0.744±0.143	0.731±0.139	0.677±0.118	0.763±0.123	Neoprot.
Cyanobia			0.972±0.157	0.968±0.153	0.868±0.133	0.983±0.134	Neoprot.
LPP basal node			2.246±0.025	2.293±0.034	2.292±0.027	2.261±0.027	Paleoprot.
LPP			1.910±0.027	2.031±0.040	1.946±0.032	1.943±0.031	Paleoprot.
Ac-Th basal node			2.105±0.023	2.204±0.036	2.219±0.027	2.113±0.027	Paleoprot.
Ac-Th			1.595±0.024	2.082±0.036	1.934±0.029	1.965±0.026	Paleoprot.
Osc-SPM-NX			2.028±0.021	2.058±0.033	2.045±0.026	1.988±0.024	Paleoprot.
Osc			1.914±0.021	1.916±0.032	1.932±0.027	1.869±0.024	Paleoprot.
SPM-NX			1.973±0.020	2.000±0.031	1.992±0.025	1.942±0.022	Paleoprot.
SPM			1.770±0.036	1.795±0.049	1.778±0.046	1.713±0.040	Paleoprot.
NX			1.918±0.018	1.935±0.027	1.933±0.022	1.887±0.019	Paleoprot.
Nostocales	1.650	2.320	1.651‡	1.651‡	1.651‡	1.651‡	Paleoprot.
Pleurocapsales+			0.646±0.099	0.689±0.101	0.680±0.108	0.622±0.089	Neoprot.
Multitrichomous filaments			1.244±0.039	1.270±0.050	1.251±0.050	1.193±0.041	Mesoprot.
False Branching			1.417±0.005	n.a.	1.426±0.013	1.418±0.006	Mesoprot.
Filamentous Sheaths^	1.900	2.450	2.331±0.024	2.293±0.034	2.140±0.027	1.988±0.024	Paleoprot.

† Ages are in Ga, error reported is one standard deviation.

‡ Estimated age abuts an age constraint, and therefore the error is near zero Ga, and the range in mean ages is the same.

+ The Pleurocapsales are not a monophyletic group; they are defined here as the clade containing *Stanieria, Xenococcus* and *Pleurocapsa* sp. PCC 7319.

∧ Character filamentous sheaths reconstructs to slightly different locations in the tree depending on the unique taxa in each tree.
